# Comprehensive phenotyping of 1,807 Indian barnyard millet (*Echinochloa frumentacea* Link) accessions from Indian national genebank: unlocking diversity for core set development

**DOI:** 10.3389/fpls.2025.1644491

**Published:** 2025-09-23

**Authors:** Badal Singh, Sushil Pandey, S. Nivedhitha, Neelam Shekhawat, Mamta Singh, Balram Jat, Chithra Devi Pandey, D. P. Semwal, Lalit Arya, R. K. Gautam, G. P. Singh

**Affiliations:** ^1^ Division of Germplasm Evaluation, ICAR-NBPGR, New Delhi, India; ^2^ Division of Germplasm Conservation, ICAR-NBPGR, New Delhi, India; ^3^ ICAR-NBPGR RS, Hyderabad, India; ^4^ ICAR-NBPGR RS, Jodhpur, Rajasthan, India; ^5^ Division of Plant Exploration and Germplasm Collection, ICAR-NBPGR, New Delhi, India; ^6^ Division of Genomic Resources, ICAR-NBPGR, New Delhi, India; ^7^ Director, ICAR-NBPGR, New Delhi, India

**Keywords:** barnyard millet germplasm, phenotyping, core set, genetic diversity, Echinochloa frumentacea

## Abstract

**Introduction:**

A comprehensive characterization of 1,807 barnyard millet (*Echinochloa frumentacea* Link.) accessions conserved in the Indian National Genebank (INGB) was conducted to assess genetic variability and develop a representative core set.

**Methods:**

Thirteen qualitative and ten quantitative traits were evaluated. Five core sets were created using Core Hunter 3, utilizing optimization approaches to enhance representativeness and diversity. Comparisons were made between the entire collection and the developed core sets using diversity indices, statistical parameters, correlation analysis, and principal component analysis (PCA).

**Results:**

Significant diversity was revealed across traits. Predominant qualitative traits included pyramidal inflorescence shape (89%), green inflorescence color (57%), and intermediate inflorescence compactness (46%). Plant height ranged from 72.36 to 213.96 cm, inflorescence length from 6.73 to 35.65 cm, and 1000-seed weight from 1.01 to 5.55 g, demonstrating a wide range of quantitative characteristics. High heritability values (82.08–94.42%) and substantial genetic advances highlighted their agronomic importance. Among the five cores, core set-3 comprising 271 accessions achieved the best balance of genetic diversity, trait representativeness, and low redundancy, with a variable rate of coefficient of variance (VR) of 110.41%, coincidence rate (CR) of 85.97%, and mean difference percentage (MD) of 30%. Shannon–Weaver diversity indices and evenness values confirmed superior diversity representation. Comparisons showed non-significant differences in means, variances, and frequency distributions for most traits between the core and entire collection. Correlation and PCA confirmed conservation of trait associations and genetic structure, with the first five principal components explaining 74.9% of total variance in the core set, closely aligning with the entire collection (70.8%).

**Conclusion:**

This study highlights the utility of the INGB barnyard millet core set as a valuable genetic resource for breeding programs. The core set provides opportunities for the effective use and preservation of barnyard millet genetic resources by improving access to genetically diverse and agronomically significant germplasm.

## Introduction

Barnyard millet (*Echinochloa frumentacea* Link), locally known as Sanwa in Hindi and Shyama in Sanskrit, is an ancient and resilient crop that is primarily cultivated in warm and temperate regions, particularly in Asia, that includes China, Japan, Korea, and India. Barnyard millet, which is the fourth most widely consumed minor millet in the world, contributes significantly to food security, especially for communities with limited resources. Its short growth cycle, ability to thrive in adverse environmental conditions and tolerance to both biotic and abiotic stresses, makes it an invaluable crop for marginal agro-ecologies, providing sustenance in areas vulnerable to climate change (Singh et al., 2010; Padulosi et al., 2009).

Barnyard millet is well known for its high nutritional content. It is abundant in dietary fiber, protein, carbohydrates and key micronutrients including zinc (Zn) and iron (Fe), all of which are critical for human health (Saleh et al., 2013; Chandel et al., 2014). These attributes make it an affordable and nutritious alternative to more commonly grown cereals, particularly for subsistence farming and as a backup during monsoon failures (Gupta et al., 2009). In India, barnyard millet serves both as a human food source and as livestock feed, and it grows well in two primary agro-ecological zones: the southern Deccan Plateau and the mid-hills of Uttarakhand in the Himalayan region. Furthermore, the crop also grows as a weed (*Echinochloa colona*) in rice fields during droughts, providing an emergency food source (Padulosi et al., 2009). In recognition of its nutritional and ecological importance, the United Nations declared 2023 the International Year of Millets to promote their global cultivation and utilization.

In addition to its many health benefits, barnyard millet is prized for its ability to fight cancer, diabetes, heart disease, obesity, skin issues, and celiac disease. It is a great dietary choice for people with diabetes and celiac disease because it contains gluten-free flour (Bhatt et al., 2023). Barnyard millet is very important in industry because of its remarkable nutritional profile. It can be used to create a variety of millet-based food products, including porridge, baby food, bread, snacks, millet wine, fast food, and nutritional powders. In addition to increasing its attractiveness to consumers who are health-conscious, this adaptability creates opportunities for its application in the nutraceutical and functional food sectors.

Barnyard millet is primarily a self-pollinating crop (some wind-mediated outcrossing) (Maun and Barrett, 1986). It includes two domesticated species: *Echinochloa frumentacea* (Indian barnyard millet) and *Echinochloa esculenta* (Japanese barnyard millet). The two species can be distinguished based on panicle morphology and spikelet characteristics. Indian barnyard millet (*E. frumentacea*) was domesticated from *E. colona* in India, while Japanese barnyard millet (*E. esculenta*) was derived from *E. crusgalli*, domesticated in Japan around 4000 year ago (Yabuno, 1971; De Wet et al., 1983; Yabuno, 1987). However, distinguishing between the domesticated, wild and weedy forms of *E. frumentacea* remains a challenge due to limited taxonomic keys and a lack of detailed understanding (Hoste and Verloove, 2022). Although the Indian National Genebank (INGB) holds both wild and cultivated varieties of *E. frumentacea* Link and *E. colona* (L.) Link, it is challenging to accurately identify these forms without conducting in-depth taxonomic research. As such, this study focuses on *E. frumentacea* and its wild and weedy relatives distributed across India, as the INGB primarily conserves accessions of this species.

Despite its significant role, barnyard millet has received limited attention in crop improvement programs, especially in India, where only a few improved varieties are available. Systematic efforts in genetic resource management are necessary to realize its potential for broader cultivation and use. About 2,000 barnyard millet accessions are conserved in India’s National Genebank at ICAR-National Bureau of Plant Genetic Resources, New Delhi. These accessions are essential genetic resources for breeding initiatives that aim to create more nutritional, stress-resistant and high-yielding cultivars. Previous studies have attempted to characterize subset of barnyard millet germplasm to explore variability in agronomic and quality traits. For instance, Joshi et al. (2015) characterized landraces for agro-morphological traits and disease resistance, while Patil et al. (2021) analyzed genetic divergence among 41 genotypes, forming twelve clusters based on yield and nutritional traits. Additionally, Prabu et al. (2020a) and Prabu et al. (2020b) worked on 40 genotypes under high altitude condition and reported substantial diversity and stable genotypes with respect to early maturity and yield performance.

The necessity of creating core collections that optimize genetic diversity while maintaining the germplasm’s representativeness has been underlined by earlier research. Although molecular marker-based studies such as Wallace et al. (2015) have provided insights into global barnyard millet diversity, they may not fully capture the extent of agro-morphological variation. Phenotypic core sets remain crucial for capturing agronomic variation and observable adaptation traits, which are particularly important in underutilized crops. Compared to molecular-based sets, phenotypically constructed core sets enable direct identification of traits of breeding interest, such as early flowering, plant stature, or grain quality. Some researchers advocate for including rare alleles and extreme traits from secondary and tertiary gene pools, such as wild and weedy relatives, in core collections (Gowda et al., 2009; Upadhyaya et al., 2014). However, there are frequently issues with the genetic resources in genebanks, including taxonomic misidentification, duplication and missing information, especially in crops like barnyard millet that have not undergone a lot of taxonomical research. The objective of this study is to create a core collection of barnyard millet that is inclusive of rare alleles, minimally redundant, and genetically representative in light of these difficulties. These core collections are necessary for efficient management and use of genetic resources, which supports attempts for better crops.

Most existing core or mini-core sets in millets, including those reported by Upadhyaya et al. (2014), have been developed from subsets of available collections, often representing only 10–20% of the total germplasm. Such limited sampling may overlook rare or geographically restricted variants. In contrast, the present study utilizes the entire conserved collection of barnyard millet at NBPGR (National Bureau of Plant Genetic Resources) to develop a phenotypically representative core set that is inclusive, minimally redundant, and genetically broad-based. This will be accomplished by doing a thorough phenotypic characterization of 1,807 barnyard millet accessions, including native wild/weedy forms conserved in the Indian National Genebank. Assessing genetic diversity, identifying critical features for crop improvement and creating a core collection that maximizes the use of these resources are the key objectives. By including wild and weedy forms that contribute valuable agronomic traits, the core set will reflect the full spectrum of genetic diversity, supporting the genetic enhancement of barnyard millet for future agricultural challenges.

## Material and methods

**Figure 1:**
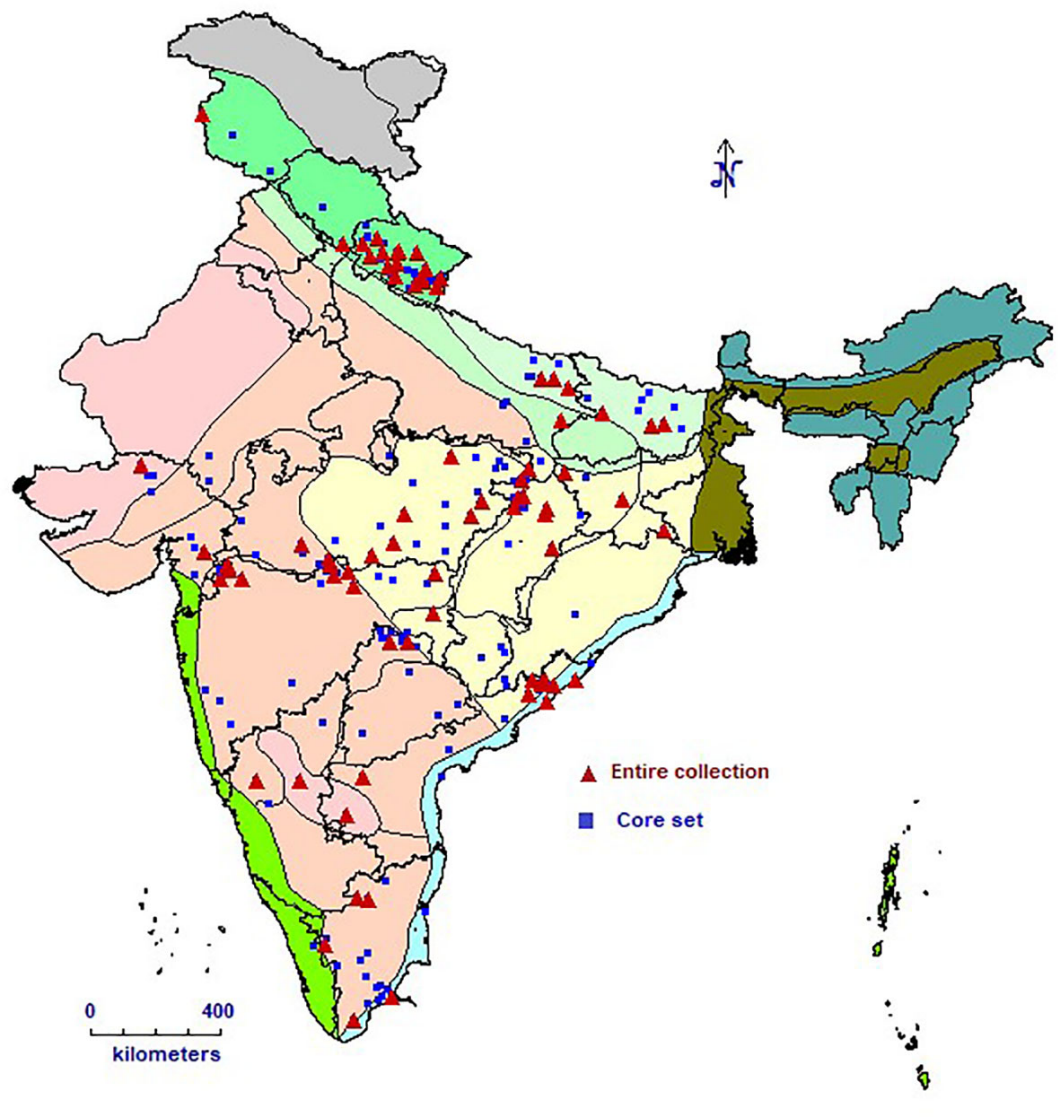
Geographical distribution and collection sites of INGB barnyard millet collection.

This study included 1,888 barnyard millet accessions with a wide geographic range (**Figure 1**), including 4 exotic collections from the USA and 1807 indigenous collections collected from several Indian states. The experiments were sown in the ICAR-NBPGR experimental farm at Issapur, New Delhi, on July 21, 2023, and at Kanha Shanti Vanam, Hyderabad, in the latter part of August 2023. Of the 1,888 accessions, data was generated for 1,807 accessions (**Table 1**) at New Delhi and 1,403 accessions at Hyderabad, as some accessions failed to germinate. The ICAR-NBPGR Issapur farm is situated 218 meters above mean sea level at latitude 28°57′ N and longitude 76°84′ E. The climate of the area is semi-arid subtropical, with 400 mm of precipitation on average each year. The soil has a pH of about 8 and varies from sandy loam to loamy sand. Kanha Shanti Vanam, Hyderabad is located at a latitude of 17°12’ N, longitude of 78°13’ E, and an altitude of 229 meters above mean sea level. The region has a semi-arid subtropical climate, with an average annual precipitation of 800 mm. The pH of the soil is roughly 6.8, and it varies from sandy loam to loamy sand. Each accession was planted in two rows, each 2 meters long, with 45 cm spacing between rows and 10 cm spacing within rows. An augmented block design with ten blocks was implemented in the experiment. There were 205 accessions in each block, and the final block was incomplete. Four checks representing leading barnyard millet varieties viz, VL207, VL172, DHBM 93-1, and Gujarat Banti 1 were randomly replicated in each block. Two stages of fertilizer application were carried out: 50% of the nitrogen and all of the phosphorus were administered during sowing, and the remaining 50% was top-dressed following the initial irrigation. For crop establishment, pre-sowing irrigation was offered. The first irrigation was given during active tillering (25–35 DAS), and the second irrigation was given during the flowering stage (35–70 DAS). Other recommended agronomic practices were adhered to, at different phases of crop development. Harvesting and manual threshing was done for every accession. Furthermore, during the 2024–2025 cropping season, a chosen group of 271 different accessions that showed superiority for a number of agronomic and phenological variables were further confirmed at two locations viz, ICAR-NBPGR, Issapur Farm, Delhi and ICAR-NBPGR, Regional Station Jodhpur. With entries dispersed throughout blocks, including the previously indicated checks, these accessions were examined using an Augmented Block Design.

**Table 1:** Information of accessions based on their passport data.

S. no	Entire collection (EC)		Core set (CS)	
	Species	Accession	Species	Accession
1.	*Echinochloa frumentacea*	1689	*Echinochloa frumentacea*	250
2.	*Echinochloa colonum*	85	*Echinochloa colonum*	13
3.	*Echinochloa crus-galli*	15	*Echinochloa crus-galli*	5
4.	*Echinochloa esculenta*	3	*Echinochloa esculenta*	0
5.	*Echinochloa oryzoides*	3	*Echinochloa oryzoides*	1
6.	Unknown	12	Unknown	2
	Total	1807	Total	271

### Phenotyping of phenological and agro-morphological characteristics

**Figure 2:**
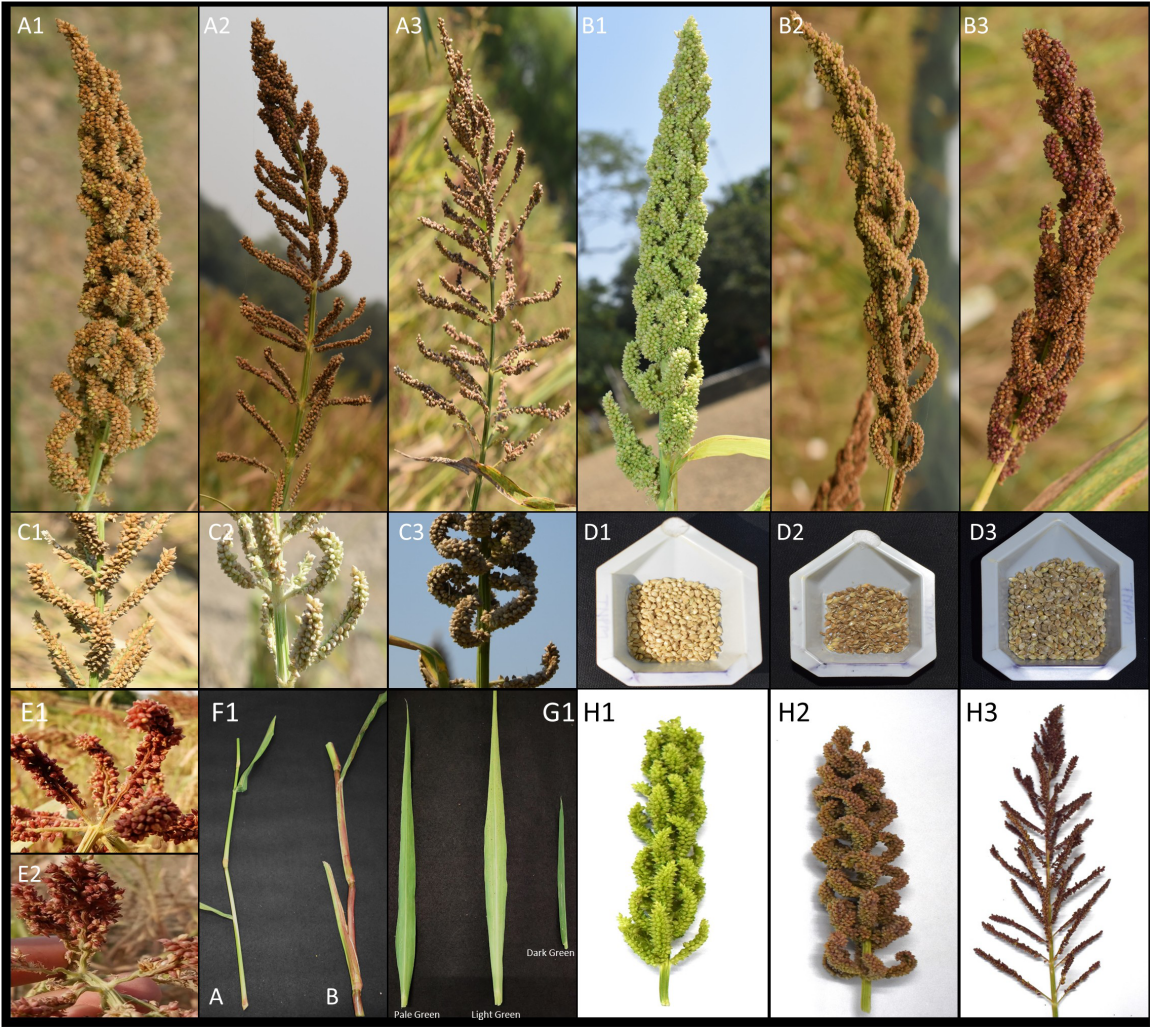
Picture representing the descriptors used in characterization of INGB barnyard millet collection. **A** (Inflorescence compactness): A1, Compact; A2, Intermediate; A3, Open. **B** (Inflorescence Colour): B1, Green; B2, Light Purple; B3, Dark Purple. **C** (Shape of Lower Raceme): C1, straight; C2, Curved; C3, Slender. **D** (Grain colour): D1, Straw White; D2, Light Grey; D3 Grey. **E** (Branching of Lower Raceme): E1, Absent; E2, Present. **F** (Plant Pigmentation): F1 A, Absent; F1 B, Present; **G** (Leaf Color), Pale green, Light Green and Dark green. **H** (Inflorescence Shape): H1, Cylinder; H2, Globose; H3, Pyramidical.

Ten quantitative and thirteen qualitative characteristics, totalling 23 traits, were assessed (**Supplementary Table 1**). The 10 quantitative traits included the total number of leaves (L), leaf length (LL, measured in cm), leaf width (LW, measured in cm), length of peduncle (LP, measured in cm), plant height (PH, measured in cm from base to tip), length of inflorescence (LI, measured in cm), grain yield per plant (GYPP, recorded in g), thousand grain weight (1000_GW, measured by weighing 1000 seeds in g), days to 50% flowering (DFLOW, recorded as the number of days from planting to 50% flowering), and basal tiller number (TN, counted per plant). The observation was taken for five plants per accession and averaged. The 13 qualitative traits were assessed visually and categorized based on distinct phenotypic expressions. These traits included growth habit, early plant vigour, leaf color, plant pigmentation, inflorescence color, inflorescence shape, inflorescence compactness, shape of the lower raceme, branching of the lower raceme, spikelet arrangement, senescence behaviour (Stay green trait), grain color, and grain shape (**Supplementary Table 1**, **Figure 2**). Although the DUS (Distinction, Uniformity and Stability) descriptors prescribed by PPVFR (Protection of Plant Variety and Farmers Right) were referred during phenotyping, they were not used directly for grouping traits into classes. Instead, the real average values of quantitative traits were recorded and used as per the **Supplementary Table 1** provided, aligning with our objective to create a core collection from the entire National Gene Bank collection with minimum redundancy. This approach ensured precise data collection while leveraging the DUS descriptors as a reference framework.

### Statistical analysis

Analysis of Variance (ANOVA) was performed on the quantitative data in accordance with Augmented Randomised Complete Block Design (Federer, 1956). The package augmentedRCBD (Aravind et al., 2019) was used in R (R Core Team, 2024) to generate adjusted means, which were then used for additional analysis. Genetic variability measures, such as the genotypic and phenotypic coefficients of variation (GCV and PCV), were computed for each characteristic (Burton, 1952). Following that, the range was categorized using Sivasubramanian and Madhavamenon’s (1978) criteria. The formula h^2^ (bs) = VG/VP (Lush, 1940) was used to determine broad-sense heritability, where VG and VP represent genotypic and phenotypic variances, respectively. It was further divided into low, medium, and high categories as per Robinson (1966). The expected genetic advance (EGA) was calculated using the formula EGA = k × √VG × h^2^ (bs), as stated by Johnson et al. (1955). The standardized selection difference (k) at 5% selection intensity is 2.06. The formula for expressing genetic progress as a percentage of mean was GA (%) = EGA mean × 100.

### Construction of the core set

For core set identification, agro-morphological and phenological trait data were normalized to remove scale discrepancies. Using the R front-end for Core Hunter 3 (CH3) software (De Beukelaer and Davenport, 2018; De Beukelaer et al., 2018), five core sets were first produced in accordance with Odong et al. (2013)’s guidelines for optimizing genetic distance-based criteria. The distance between each accession and the closest entry in the core (A-NE) and the distance between each entry and its closest neighbouring entry (E-NE), as determined by the Gower distance, were the two methods used to optimize the average genetic distances. The core sets were formed using the strategies: Maximizing E-NE distances (Core set 1), maximizing A-NE distances (Core set 2), maximizing both E-NE and A-NE with equal weight (1:1) (Core set 3), maximizing E-NE and A-NE with unequal weight (0.3:0.7) (Core set 4), and maximizing E-NE and A-NE with unequal weight (0.7:0.3) (Core set 5).

Based on their limited representation in the genebank, their potential for particular traits, and their superior performance in one or more traits seen in the field, a group of 50 accessions was chosen for inclusion in all core sets. According to the neutral allele theory, the core set size was set at roughly 15% of the entire collection (Brown, 1989).

### Analysis of the core set

Together with other statistical factors, the five core sets were assessed using the genetic distance criteria described by Odong et al. (2013). These included the “coverage” criteria for qualitative traits as outlined by Kim et al. (2007), as well as the mean difference percentage (MD%), variable rate of coefficient of variation (VR%), and coincidence rate of range (CR%) for quantitative traits, in accordance with the methodology of Hu et al. (2000). Further analyses included calculations of the Kullback-Leibler distance (Kullback and Leibler, 1951), Kolmogorov-Smirnov P-value (Smirnov, 1939) and Anderson-Darling P-value (Anderson and Darling, 1954). The correlation between the trait correlation matrix of each core set and the collection as a whole was evaluated using the Mantel test (Mantel, 1967). For additional comparative studies, the core set that showed the most diversity and representativeness was chosen. In particular, the core representing the extreme values of the attributes was compared using the Anderson-Darling P-value. Using the Newman-Keuls test (Newman, 1939; Keuls, 1952) and the t-test, the quantitative trait means of the chosen core set and the complete collection were compared. Levene’s test (Levene, 1960) was used to confirm that the variances in the entire collection and the chosen core set were homogeneous, and the Wilcoxon rank test (Wilcoxon, 1945) was used to assess variations in frequency distribution. The frequency distributions of the core set and the entire set were visualized and compared using boxplots, and the variations in continuous trait distributions were presented overall using quantile-quantile (QQ) plots (Wilk and Gnanadesikan, 1968). Using the phenotypic frequencies of qualitative traits, the Shannon-Weaver diversity index (H^`^) and evenness (Shannon and Weaver, 1949) were computed. In order to investigate the correlations among quantitative and qualitative characteristics in both the core set and the complete collection, Pearson correlation coefficients were determined. Relationships between traits and their contributions to multivariate variation have been identified using Principal Component Analysis (PCA). The R package EvaluateCore was used for all statistical analyses of the core sets (Aravind et al., 2020).

## Results

### Qualitative trait variability

**Figure 3:**
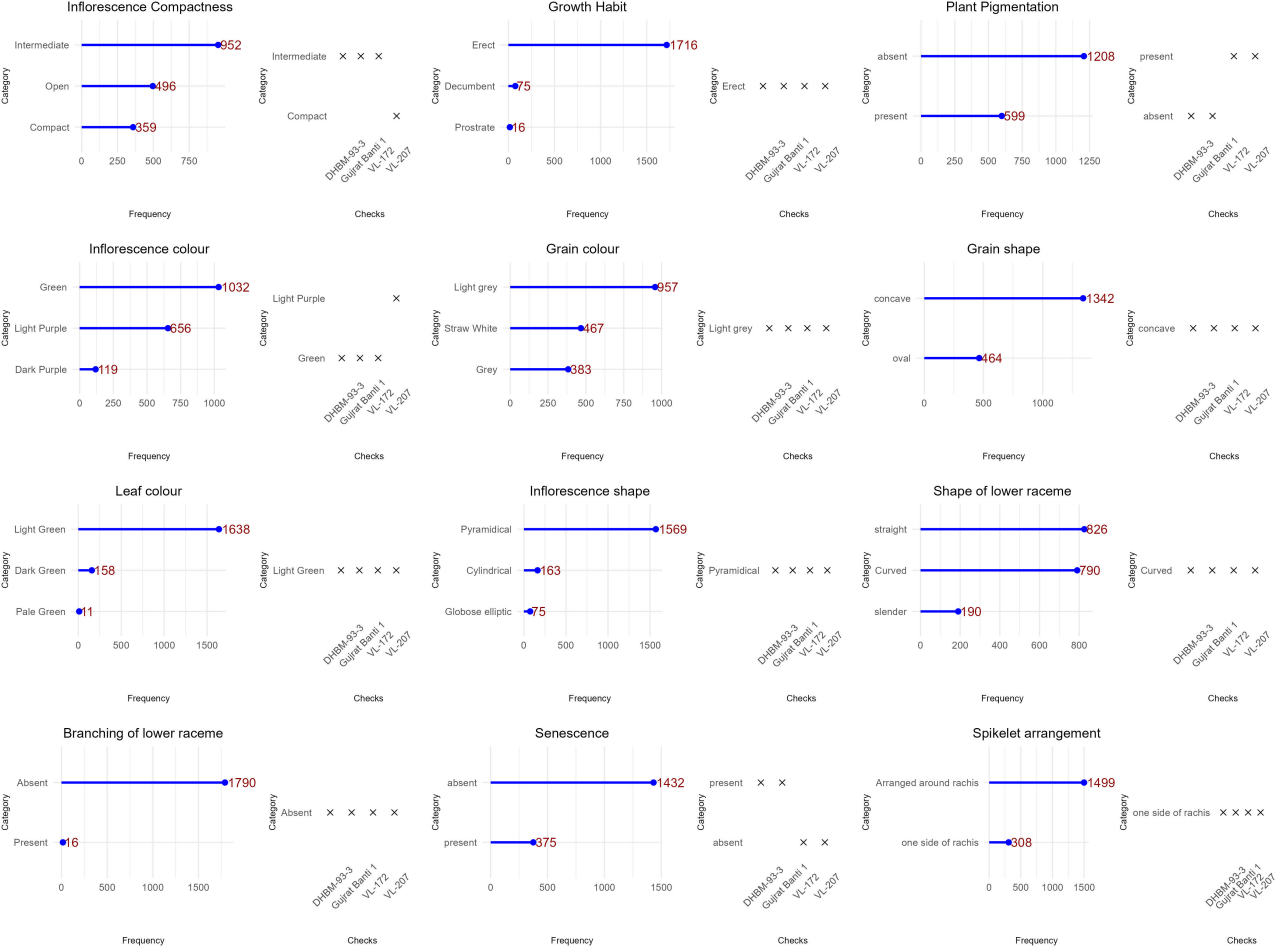
Frequency dot plot of 1807 barnyard millet accession for qualitative traits.

The 13 qualitative qualities that were assessed showed significant diversity (**Figure 3**; **Supplementary Figure 3**). The majority of accessions showed an erect growth habit (96%) and very good early vigor (97%). Two-thirds of the leaves were pigmentless, and the majority (89%) were light green in color. Inflorescence characteristics were more diverse, with the most common being pyramidal form (89%) and green color (57%) but also notable purple coloring and other morphologies. There were three different levels of compactness: open (30%), compact (24%), and intermediate (46%). Grain-related characteristics varied: the most prevalent grain shape was concave (74%), whereas the most common grain color was light gray (54%). Significant morphological heterogeneity within the collection is confirmed by these findings.

### Quantitative trait variation and genetic parameters

**Figure 4:**
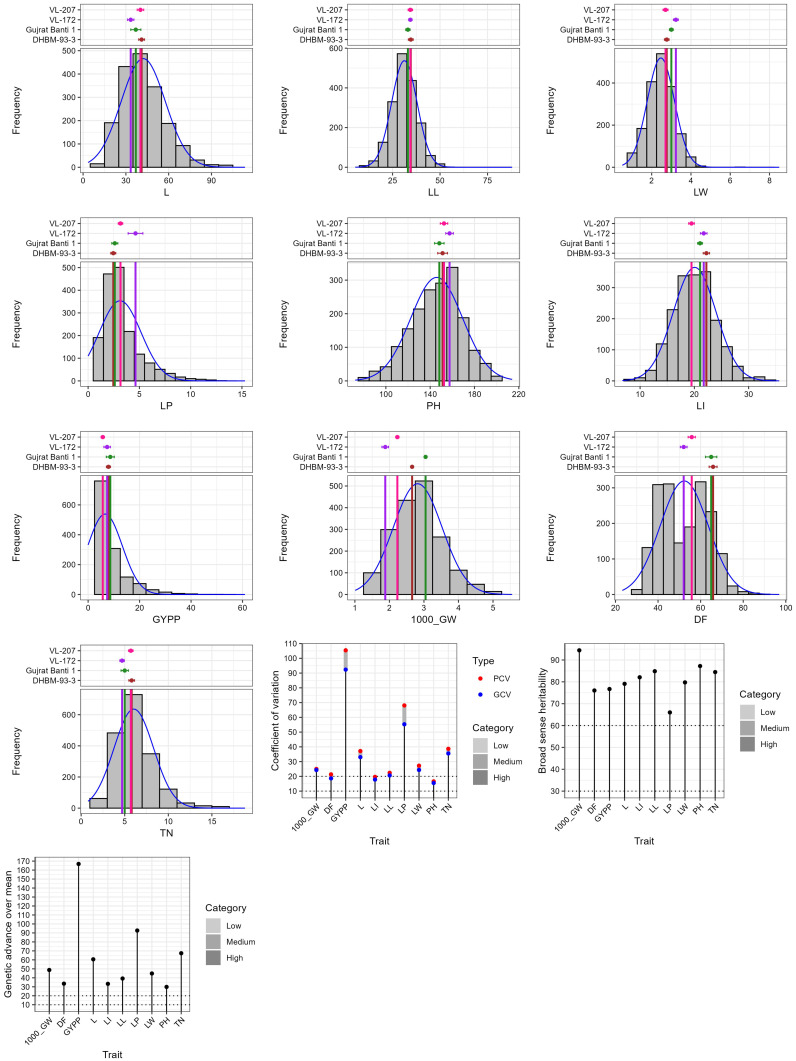
Frequency distribution plot of 1807 barnyard millet accession for quantitative traits.

Wide variation was recorded among the accessions as evident from frequency distribution plot for the 10 quantitative traits (**Figure 4**) and general statistical analysis (**Table 2**). The total number of leaves (L) ranged from 3.51 to 113.51 with a mean of 42.16 ± 0.36. Leaf length (LL) showed a range of 5.35 to 87.96 cm, with a mean of 31.21 ± 0.16 cm. Leaf width (LW) exhibited moderate variability with a range of 0.51 to 8.50 cm and a mean of 2.47 ± 0.02 cm. The length of the peduncle (LP) ranged from 0.00 to 15.30 cm, with a mean of 3.12 ± 0.05 cm. Plant height (PH) ranged from 72.36 to 213.96 cm, with an average of 145.88 ± 0.55 cm. Similarly, length of inflorescence (LI) ranged from 6.73 to 35.65 cm, with a mean of 20.06 ± 0.09 cm and high heritability (82.08%), suggesting a strong genetic basis for the trait. Grain yield per plant (GYPP) exhibited the greatest variability among all traits, ranging from 0.00 to 60.96 g with a mean of 6.58 ± 0.16 g. Thousand grain weight (1000_GW) ranged from 1.01 to 5.55 g, with a mean of 2.82 ± 0.02 g. Days to 50% flowering (DFLOW) ranged from 23.23 to 96.92, with a mean of 52.31 ± 0.27 days. Finally, basal tiller number (TN) varied from 0.80 to 18.80, with a mean of 6.04 ± 0.05. Overall, traits such as GYPP, LP, TN, and 1000_GW demonstrated high variability coupled with high heritability and genetic advance, indicating their utility for selection and genetic improvement. In contrast, traits like PH and DFLOW showed moderate variability and high heritability, suggesting consistent genetic control with moderate potential for improvement.

**Table 2:** General statistical parameters and genetic diversity estimates for 1809 barnyard millet germplasm accessions.

Trait	Min	Max	Mean ± SE	GCV	PCV	H^2^ (BS)	GA	GAM
L	3.51	113.51	42.16 **±** 0.36	33.03	37.14	79.09	25.55	60.60
LL	5.35	87.96	31.21 **±** 0.16	20.64	22.40	84.85	12.24	39.22
LW	0.51	8.50	2.47 **±** 0.02	24.33	27.24	79.74	1.11	44.82
LP	0.00^a^	15.30	3.12 **±** 0.05	55.29	68.06	66.00	2.89	92.67
PH	72.36	213.96	145.88 **±** 0.55	15.50	16.60	87.22	43.56	29.86
LI	6.73	35.65	20.06 **±** 0.09	17.81	19.66	82.08	6.68	33.28
GYPP	0.00^a^	60.96	6.58 **±** 0.16	92.33	105.42	76.71	10.98	166.83
1000_GW	1.01	5.55	2.82 **±** 0.02	24.30	25.01	94.42	1.37	48.71
DFLOW	23.23	96.92	52.31 **±** 0.27	18.62	21.35	76.04	17.52	33.49
TN	0.80	18.80	6.04 **±** 0.05	35.54	38.67	84.46	4.07	67.38

L, Total number of leaves; LL, Leaf length (cm); LW, Leaf width (cm);LP, length of peduncle (cm); PH, Plant height (cm); LI, length of inflorescence (cm); GYPP, Grain yield per plant (g); 1000_GW, Thousand grain weight (g); DFLOW, Days to 50% Flowering; TN, Basal Tiller number; GCV, genotypic coefficient of variation; PCV, phenotypic coefficient of variation, h2 (bs), broad-sense heritability; GA, genetic advance; GAM, genetic advance over mean. ^a^Negative adjusted mean values were considered as 0.

### Identification of trait-specific accessions

Evaluation of genetic resources for identification of trait-specific promising donors is important for enhancing the utilization and strategic management of conserved germplasm. To support the strategic use of conserved germplasm, we identified promising trait-specific accessions based on consistent performance across four environments—Delhi (2023–24), Hyderabad (2023–24), Delhi (2024–25), and Jodhpur (2024–25) (**Table 3**, **Supplementary Table 2**). These accessions exhibit trait values that surpass those of the best-performing checks, indicating their potential as donor lines in targeted breeding programs.

**Table 3:** Trait specific promising germplasm identified in barnyard millet.

Trait	Promising accessions across all locations (mean value^*^)	Checks (mean value*)
Plant Height (cm)	IC0472451 (56.86 cm), IC0589379 (60.44 cm), IC0472320 (64.33 cm)	Gujrat Banti 1 (125.31 cm), DHBM-93-3 (118.65 cm), VL-207 (120.18 cm), VL-172 (120.70 cm)
Length of Inflorescence (cm)	IC0279608 (31.65 cm), IC0472576 (29.03 cm)	Gujrat Banti 1 (19.33 cm), DHBM-93-3 (19.27 cm), VL-207 (17.91 cm), VL-172 (20.01 cm)
Thousand Grain Weight (g)	IC0306390 (4.71 g), IC0404359 (4.60 g)	Gujrat Banti 1(2.89 g), DHBM-93-3 (2.93 g), VL-207 (2.74 g), VL-172 (2.70 g)
Days to 50% Flowering	IC0404530 (35 days), IC0426592 (36 days), IC0404300 (37 days), IC0340124 (35 days)	Gujrat Banti 1 (63 days), DHBM-93-3 (62 days), VL-207 (56 days), VL-172 (51 days)
Basal Tiller Number	IC0041791 (12), IC0624707 (13), IC0601265 (14)	Gujrat Banti 1 (3), DHBM-93-3 (4), VL-207 (4), VL-172 (3)

* Data represent mean performance across four environments: Delhi (2023–24), Hyderabad (2023–24), Delhi (2024–25), and Jodhpur (2024–25).

### Core set development and quality assessment

Five core sets were developed using Core Hunter 3 and evaluated for diversity and representativeness (**Table 4**). Core set-1 exhibited the highest diversity measures, including E-NE (0.068), E-E (0.174), and H’ (1.01), indicating a strong capture of genetic variation. In contrast, Core set-2 showed the lowest values for E-NE (0.055) and E-E (0.136), reflecting limited diversity. Core set-1 also had the highest CR% (93.56%) and VR% (117.98%), demonstrating effective trait coverage. Meanwhile, Core set-2 had the lowest CR% (80.65%) and VR% (97.71%). In terms of redundancy, Core set-2,3 and 4 had the lowest A-NE (0.048), suggesting minimal duplication in accessions. Core sets-1 and 5 exhibited relatively higher MD% (50% and 40%, respectively), while Core sets-2 and 4 had the lowest MD% (20%). All core sets, except Core set-2, achieved full class coverage, ensuring comprehensive representation. Core set-5 had the smallest Kullback-Leibler (KL) distance (0.06), indicating closer alignment with the collection’s distribution. Core sets-3 and 4 performed best in distribution tests, with most traits showing high p-values (K-S > 0.01, A-D > 0.01), signalling better trait distribution similarity to the entire collection. Mantel correlations showed strong genetic relationships, with values ranging from 0.953 for Core set-2 to 0.977 for Core set-1, indicating strong conservation across core sets. Based on these indices, Core set-1 emerged as the most diverse and representative, while Core set-3 (hereafter referred to as INGB barnyard millet core set) was noted for having low redundancy and strong performance in trait distribution, making it a strong alternative for applications requiring a balance between diversity, representativeness and capturing of extreme traits.However, core set-3, hereafter referred to as the INGB (Indian National Gene Bank) core set, was selected as the final core due to its balance of diversity, low redundancy, and superior trait distribution performance. It consists of 271 accessions from multiple *Echinochloa* species, broadly representing the geographical (**Figure 1**) and biological diversity of the full collection (**Table 1**; **Supplementary Table 3**).

**Table 4:** Comparison of different core sets developed based on core quality evaluation indices.

Criteria	Core Set 1	Core Set 2	Core Set 3	Core Set 4	Core Set 5
E-NE	0.068	0.055	0.063	0.060	0.066
A-NE	0.049	0.048	0.048	0.048	0.049
E-E	0.174	0.136	0.160	0.152	0.169
MD%	50	20	30	20	40
Class coverage	100	97.4359	100	100	100
CR%	93.56	80.65	85.97	86.54	92.51
VR%	117.98	97.71	110.41	106.14	116.33
Mantel correlation	0.977**	0.954**	0.973**	0.964**	0.967**
Average H	1.01	0.87	0.98	0.94	0.99
Average KL	0.08	0.16	0.09	0.11	0.06
Percentage of Traits with K-S (P-value > 0.01)	60	80	80	90	60
Percentage of Traits with A-D (P-value > 0.01)	30	70	70	80	50

E-EN, the average distance between each entry and nearest neighboring entry; A-NE, the average distance between each accession and the nearest entry; E-E, the average genetic distance between entries; MD%, Mean difference percentage; CR%, coincidence rate of range; VR%, variable rate of range; H’, Shannon diversity index; KL, Kullback-Leibler Distance; KS, Kolmogorov-Smirnov Distance; A-D, Anderson-Darling Distance **indicates significance at p = 0.001. Bold letter is selected core set.

### Comparison with entire collection

**Figure 5:**
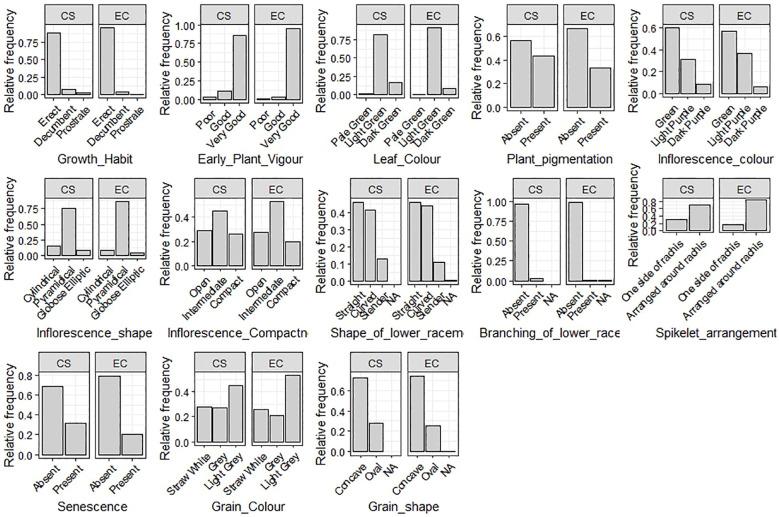
Relative frequency plot showing comparison of variability of qualitative traits in the Entire collection (EC) and core set (CS) of barnyard millet.

**Figure 6:**
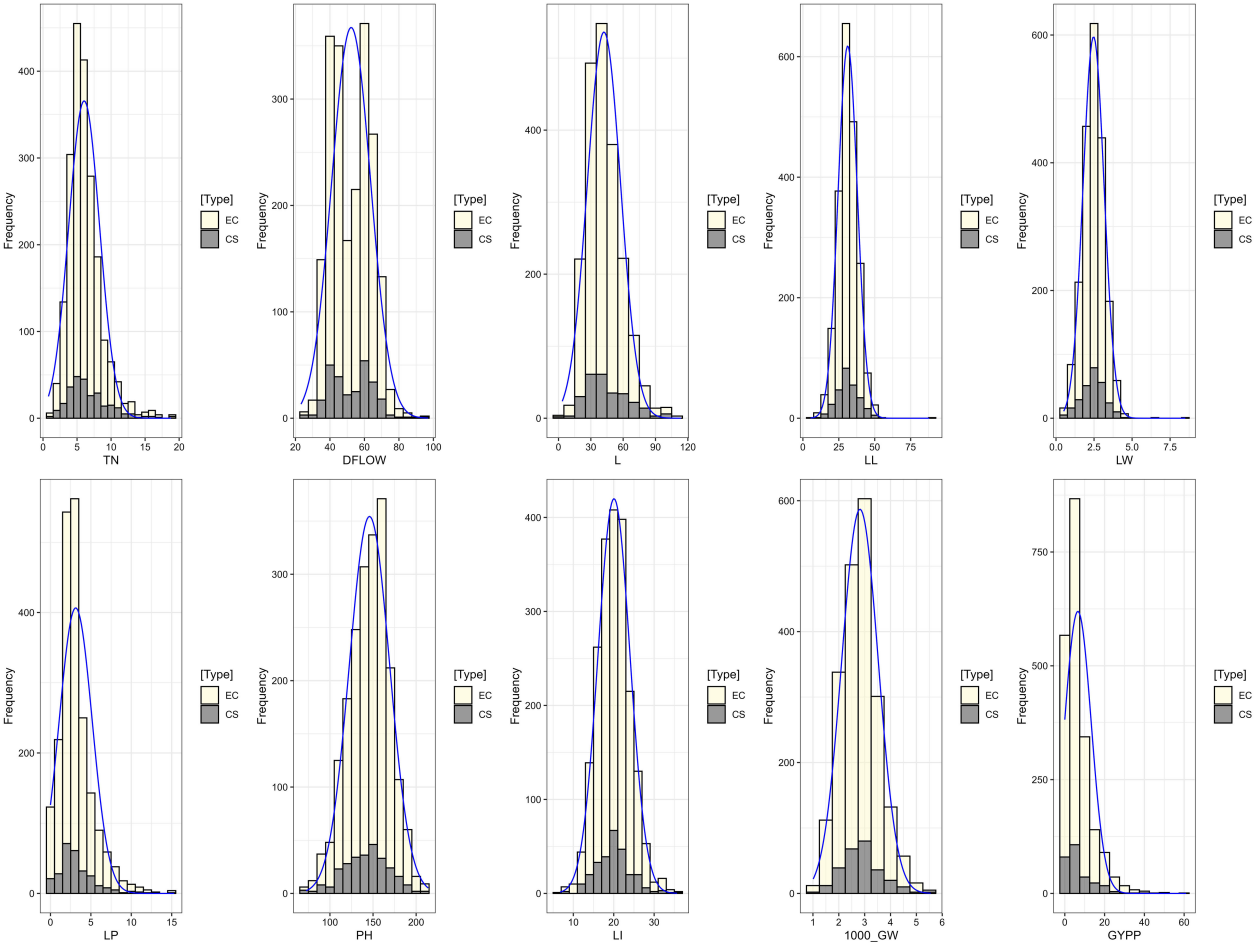
Frequency distribution plot showing comparison of variability of quantitative traits in the entire collection (EC) and core set (CS) of barnyard millet.

The INGB core set represent the overall diversity of entire collection with respect to qualitative as well as quantitative traits. The Shannon-Weaver diversity index (H’) for qualitative traits (**Table 5**) showed that the core set, which is demonstrated as well in **Figure 5**, maximized the diversity of features contained in the overall collection. For every characteristic, the core set’s evenness values were consistently higher. These higher evenness values imply that the diversity found in the total collection is more accurately represented by the core set.The means, ranges, coefficients of variation (CV), interquartile ranges, and frequency distributions for a variety of quantitative features in the chosen core set and the entire collection are shown in **Table 6** and are depicted in **Figure 6**. The higher coefficients of variation (CV) for all traits in the core set indicated greater variability in the quantitative traits compared to the full collection. Except for traits like TN, L and PH, differences in means between core and entire set was found non-significant (1% probability level) as confirmed by both the Newman-Keuls and t-tests. However, L is non-significant at 5% probability level (t test) and PH is non-significant at 5% probability level (Newman-keuls test, t test). Similarly with respect to difference in variance between core and entire set, non-significant difference is found for majority of traits (except TN, L, LP and PH) as confirmed by Levene’s test. The Wicoxon rank test, which verified the difference in frequency distribution between the core and the entire set, showed non-significant differences for all traits (TN and PH excluded). **Supplementary Figure 1** and **2** show a boxplot and a qqplot that illustrate the variation of quantitative features in the core set (CS) and the entire collection (EC).

**Table 5:** Shannon diversity index of qualitative traits in the entire collection and core set of barnyard millet germplasm.

Traits	Shannon weaver diversity index (H’)		H’ max		Evenness	
	EC	CS	EC	CS	EC	CS
Growth Habit	0.32	0.63	1.1	1.1	0.29	0.57
Early Plant Vigour	0.31	0.71	1.1	1.1	0.28	0.64
Leaf Colour	0.48	0.74	1.1	1.1	0.44	0.68
Plant pigmentation	0.92	0.98	0.69	0.69	1.32	1.42
Inflorescence colour	1.25	1.29	1.1	1.1	1.14	1.18
Inflorescence shape	0.68	1.06	1.1	1.1	0.62	0.97
Inflorescence Compactness	1.46	1.54	1.1	1.1	1.33	1.41
Shape of lower racemes	1.38	1.42	1.1	1.1	1.26	1.29
Branching of lower racemes	0.07	0.19	0.69	0.69	0.11	0.28
Spikelet arrangement	0.66	0.88	0.69	0.69	0.95	1.27
Senescence	0.74	0.88	0.69	0.69	1.06	1.28
Grain Colour	1.46	1.55	1.1	1.1	1.33	1.41
Grain shape	0.82	0.85	0.69	0.69	1.19	1.23

**Table 6:** Comparison of range, mean, coefficient of variation, inter-quantile range, and frequency distribution in the entire collection and core set for various quantitative descriptors used in the formation of core collection in barnyard millet.

Traits	Entire collection					Core set					Difference between core set and entire collection			
	Min	Max	Mean ± SE	CV	Inter-quartile range	Min	Max	Mean± SE	CV	Inter-quartile range	Mean^a^	Mean^b^	Variance^c^	Frequency distribution^d^
TN	0.80	18.80	6.04	0.38	2.25	0.80	18.80	6.66	0.46	3.25	**	**	**	**
DFLOW	23.23	96.92	52.30	0.22	19.25	23.23	96.91	52.48	0.22	19.43	ns	ns	ns	ns
L	3.51	113.51	42.16	0.37	19.62	3.51	104.01	45.15	0.43	27.87	**	*	**	ns
LL	5.34	87.96	31.21	0.21	8.22	12.07	52.31	31.29	0.23	9.16	Ns	ns	ns	ns
LW	0.51	8.50	2.47	0.28	0.89	0.50	4.28	2.44	0.30	0.92	Ns	ns	ns	ns
LP	^a^0.0	15.30	3.12	0.65	1.97	0.00	15.30	3.23	0.73	2.31	Ns	ns	*	ns
PH	72.36	213.96	145.88	0.16	31.69	72.36	212.97	142.32	0.19	36.32	*	*	**	*
LI	6.73	35.65	20.06	0.20	5.09	7.89	35.65	20.00	0.22	5.19	Ns	ns	ns	ns
1000_GW	1.01	5.55	2.82	0.25	0.91	1.15	5.55	2.89	0.25	0.88	Ns	ns	ns	ns
GYPP	^a^0.0	60.96	6.59	1.01	6.3	0.0	49.68	6.79	1.06	6.77	Ns	ns	ns	ns

L, Total number of leaves; LL, Leaf length (cm); LW, Leaf width (cm);LP, length of peduncle (cm); PH, Plant height (cm); LI, length of inflorescence (cm); GYPP, Grain yield per plant (g); 1000_GW, Thousand grain weight (g); DFLOW, Days to 50% Flowering; TN, Basal Tiller number; ^a^Negative adjusted mean values were considered as 0. ^a^Difference between means of core set and entire collection were tested by Newman-Keuls test; ^b^ Difference between means of core set and entire collection were tested by t-test; ^c^Variance homogeneity as tested by Levene’s test.; ^d^ Difference of frequency distribution by Wicoxon rank test.

Ns indicate not significant; * and ** indicate significant differences at 5% and 1% probability level.

### Trait associations and multivariate structure

Correlation analysis showed consistent trait relationships in both the core and entire sets (**Supplementary Table 4**; **Supplementary Figure 4**). Notably, TN and L were strongly correlated, and plant height correlated positively with several other growth traits. Principal Component Analysis (PCA) revealed similar multivariate structure in both datasets, with five principal components explaining ~70–75% of total variance (**Table 7**; **Supplementary Figure 5**, **Supplementary Table 5**). Key traits contributing to variance were consistent across the core and full sets, confirming the structural integrity of the selected core.

**Table 7:** Comparison of first five principal components in entire collection and core set of barnyard millet germplasm.

Principal components	Entire collection			Core set		
	Standard deviation	Proportion of variance	Cumulative proportion	Standard deviation	Proportion of variance	Cumulative proportion
PC1	1.503312	0.225990	0.225990	1.558747	0.242970	0.242970
PC2	1.332679	0.177600	0.403600	1.369454	0.187540	0.430510
PC3	1.070854	0.114670	0.518270	1.102683	0.121590	0.552100
PC4	1.019468	0.103930	0.622200	0.997416	0.099480	0.651580
PC5	0.9253451	0.0856300	0.7078300	0.9438662	0.0890900	0.7406700

## Discussion

### The INGB barnyard millet collection’s phenotypic diversity

This study represents the first comprehensive phenotyping of the INGB barnyard millet collection, a significant advancement over previous studies that focused on fewer accessions. Early plant vigour was strong across most accessions, consistent with findings from Nandini et al. (2016). The growth habit was mostly erect, with only a few accessions showing decumbent or prostrate tendencies, as also noted by Vikram et al. (2020). Light green leaf color was most common, with a few accessions displaying darker or pale green, in line with Sood et al. (2016). Pigmentation varied, with non-pigmented plants more prevalent, a trend observed by Joshi et al. (2015). Inflorescence color was mainly green, with some accessions showing lighter purple shades, reflecting findings by Jyothi et al. (2020). Most accessions had pyramidical inflorescences, with some showing cylindrical and globose elliptical shapes, as reported by Prabu et al. (2020a). Intermediate inflorescence compactness was most common, supported by Prabu et al. (2020b). The Stay Green trait was rare, consistent with Prasanna et al. (2013b). Lower raceme shape varied, with straight and curved types being predominant, as noted by Jyothsna et al. (2016b). Branching of the lower raceme occurred in a few accessions, in agreement with Prasanna et al. (2013a). Spikelet arrangement around the rachis was typical, as observed by Jyothsna et al. (2016c). Seed color was predominantly light grey, with fewer accessions showing straw white or grey, as documented by Rao and Agrawal (2000). Concave seed shape was more common, in line with Arya et al. (2017). Notable variability in plant height and leaf length was observed, similar to findings by Jyothsna et al. (2016b) and Prabu et al. (2020a). High genetic coefficients of variation (GCV) and heritability for traits like grain yield per plant and 1000 grain weight suggest these traits are promising for breeding, in agreement with Gohel and Chaudhari (2018) and Amarnath et al. (2018).

The identification of promising accessions based on performance across multiple environments offers a valuable resource for trait-specific breeding in barnyard millet. Accessions such as IC0472451, IC0589379, and IC0472320, which exhibited significantly reduced plant height compared to checks, are ideal candidates for developing dwarf or lodging-resistant varieties, which are crucial for improving crop stability under adverse field conditions. The exceptional inflorescence length observed in IC0279608 and IC0472576, along with superior thousand grain weight in IC0306390 and IC0404359, underscores their potential to enhance seed-bearing capacity and overall grain quality, addressing important agronomic traits for marketability. Additionally, the early-maturing accessions IC0404530, IC0426592, IC0404300, and IC0340124, which reached 50% flowering earlier than checks, could provide critical genetic resources for improving barnyard millet’s adaptation to short-duration cropping systems. The high tillering ability of IC0041791, IC0624707, and IC0601265, with 12–14 basal tillers, further demonstrates their potential for contributing to improved biomass production and yield. These trait-specific accessions hold promise for targeted breeding efforts, contributing to the development of improved ideotypes for various agro-ecological zones.

### INGB barnyard millet core: a comprehensive representation of diversity and extreme traits from the entire collection

*Ex-situ* germplasm collections have grown significantly in size and number in recent decades, making it increasingly challenging to evaluate, utilize, and conserve genetic resources. In order to overcome these problems, the idea of core collections was developed in order to maximize genetic diversity within the collection as a whole while improving evaluation and utilization efficiency (Frankel, 1984; Brown, 1989). The two main goals of core collections are (i) to maximize the overall genetic diversity within the core, which is frequently preferred by genebank curators, taxonomists, and geneticists, and (ii) to maximize the representativeness of genetic diversity, which is a top concern for plant breeders (Marita et al., 2000). Furthermore, rather than concentrating only on diversity, users are placing a greater emphasis on maximizing the possibility of finding uncommon alleles or novel accessions that are adaptable to breeding resilient genotype. Rare alleles, often associated with extreme traits, are typically found in secondary and tertiary gene pools (wild and weedy relatives), highlighting the need for classifying accessions into primary and secondary gene pools before core development. This becomes particularly relevant in the context of climate change, as breeders seek to incorporate rare alleles for enhanced biotic and abiotic stress resistance. As our goal was to develop a core set that serves both breeders and taxonomists, we focused on capturing overall diversity as well as extreme trait expressions. Unlike the core set developed by Wallace et al. (2015), which was based on genotyping-by-sequencing (GBS) of a global subset and emphasized neutral genetic diversity, our approach relied on phenotypic evaluation of the entire Indian NGB collection. While Wallace et al. (2015) successfully established SNP-based species level resolution and population structure, their core set was limited in capturing Shannon`s H`. In contrast, our core set maintained broad coverage for key traits like plant height and 1000 seed weight and achieved high Shannon-Weaver diversity indices across qualitative traits. Moreover, our set retained rare morphotypes, including wild *E. frumentacea* accessions, which are often absent from global collections, highlighting its value in representing localized,adaptive variation. While molecular markers are useful for assessing genetic variation, they may not always reflect traits of agronomic or adaptive importance. In underutilized crops like barnyard millet—where genome-wide marker data are limited and costly to generate for large collections—phenotypic data provide a practical and directly relevant basis for core set development. This approach is especially valuable for breeding programs, as it reflects real-world trait performance under field conditions.

The development and evaluation of five core sets using multiple statistical approaches revealed that Core 3 offered the best balance between genetic diversity and representativeness. It exhibited a high E-E distance of 0.160 and E-NE of 0.063, indicating a wide genetic base as recommended by Franco et al., 2006 and Thachuk et al., 2009. Additionally, Core 3 maintained a low A-NE of 0.048, supporting its strong representativeness (Odong et al., 2013). With MD% of 30—within the acceptable range of <20%–30% (Hu et al., 2000; Agrama et al., 2009)—Core 3 minimizes redundancy while retaining trait diversity. It achieved complete class coverage (100%), a CR% of 85.97, and a VR% of 110.41, exceeding standard thresholds for well-structured core sets (Hu et al., 2000; Agrama et al., 2009). These metrics underscore its suitability for breeding and germplasm management. Similar methods of core set evaluation have been applied in other crops such as Indian mustard (Nanjundan et al., 2021), wheat (Phogat et al., 2021), chickpea (Archak et al., 2016), safflower (Kumar et al., 2016), rice (Agrama et al., 2009), and barley (Kaur et al., 2022).

Core 3 preserved distribution patterns and extremes, as reflected in Kolmogorov–Smirnov and Anderson–Darling tests, which showed that 80% and 70% of traits, respectively, followed the original collection’s distribution at a 1% significance level. Although Core 2 and Core 4 had slightly lower MD%, their performance in CR% and VR% was weaker. Core 1 and Core 5, while diverse, showed higher MD% and less favourable KL distances. Core 3 also had higher coefficients of variation (CV) than the entire collection for most traits, in line with Franco et al. (2006) and Thachuk et al. (2009), indicating greater phenotypic variability. Interquartile ranges were largely consistent with the entire collection, except for leaf length and basal tiller number. Quantile-Quantile (QQ) plots (**Supplementary Figure 2**) and Kullback-Leibler (KL) distances (0.06–0.11) confirmed strong similarity between trait distributions in Core 3 and the full set (Wilk and Gnanadesikan, 1968; Kullback and Leibler, 1951).

Frequency distribution analysis (**Figure 5**, **6**; **Supplementary Figure 1**) showed that Core 3 captured the full range of qualitative and quantitative traits. Notably, diversity (Shannon’s index, H′) increased in the core for key qualitative traits such as Grain Color (1.46 to 1.55) and Inflorescence Compactness (1.46 to 1.54), while rare traits like Branching of Lower Racemes showed marked gains in both diversity (H′ 0.07 to 0.19) and evenness (0.11 to 0.28), aligning with the goal of retaining rare variants important for trait-specific improvement (Thachuk et al., 2009). Overall, Core 3 stands out as a strategically curated subset suitable for both breeding and conservation.

A strong positive correlation was observed between TN and L in both the EC (0.80) and the CS (0.82), indicating that increased tillering is associated with more leaves, contributing to greater vegetative growth and potential photosynthetic capacity. Similar associations were reported by Prabu et al. (2020b) and Jyothi et al. (2020), suggesting these traits are interconnected and valuable for selection in breeding programs. LL also showed positive correlations with LW and LI, with moderate correlations in both EC (0.42, 0.43) and CS (0.57, 0.50), aligning with observations by Rao and Agrawal (2000) and Gohel and Chaudhari (2018). These traits influence plant architecture and yield potential. PH was positively correlated with LL and LW (EC: 0.33, 0.34; CS: 0.42, 0.44), reinforcing findings by Vikram et al. (2020) and Prasanna et al. (2013a) that taller plants tend to have larger leaves. A weak negative correlation between TN and DFLOW was observed (EC: -0.14; CS: -0.10), suggesting higher tillering may be linked to delayed flowering, though this trend contrasts with Jyothsna et al. (2016b) and Amarnath et al. (2018), and may reflect environmental or genetic interactions. Minor differences in correlation strength between EC and CS were observed, such as a slightly stronger association between DFLOW and LL in CS (0.17 vs 0.16) and a more pronounced correlation between L and LL in CS (0.08 vs 0.03), indicating refined trait relationships in the core set. While 1000_GW showed weak correlations overall, its slightly stronger association with PH (0.09) and GYPP (0.13) in CS supports findings by Jyothi et al. (2020) and suggests potential for indirect selection. Overall, the core set retained most key trait relationships seen in the entire collection, with only slight variations, validating its use in breeding applications (**Supplementary Table 4**, **Figure 6**).

PCA results showed similar variance structure between the EC and core set CS, with the first five components explaining 70.8% and 74.9% of the variance, respectively. Key traits like TN and LL contributed most to PC1 in both, indicating consistent trait influence. Similar loadings in other PCs confirm that CS retained the major axes of variation (**Supplementary Table 5**). These findings align with Odong et al. (2013) and Thachuk et al. (2009), supporting the structural integrity of the CS. The present study is the first to phenotype the entire National Genebank collection of barnyard millet in India, comprising 1,807 accessions in contrast to Upadhyaya et al. (2014), who developed a mini core set based on agro-morphological traits from a subset of 736 accessions in the ICRISAT collection, using a different base population and selection environment.

The results clearly highlight a rich diversity in agro-morphological and phenological traits within our core set which is derived from a broader and more comprehensive germplasm base which is effectively preserved in the extracted core set., capturing greater phenotypic diversity than previous efforts. This underscores the vital role of agro-morphological characterization in unlocking genetic variability for strategic germplasm conservation and optimal utilization, even amidst shrinking overall genetic diversity.

## Conclusion

The comprehensive characterization of 1,807 barnyard millet accessions revealed remarkable variability across 10 qualitative and 13 quantitative traits, underscoring the genetic richness within this crop. From this diversity, a core set was meticulously extracted, effectively capturing the spectrum of variability while ensuring representativeness, minimal redundancy, and inclusion of extreme traits. This core set stands as a valuable resource for advancing crop improvement initiatives, particularly for breeding climate-resilient varieties. Additionally, it provides a solid foundation for genome-wide association studies, paving the way to uncover the genetic underpinnings of key traits and accelerating progress in harnessing barnyard millet’s full potential.

## Data Availability

The original contributions presented in the study are included in the article/**Supplementary Material**. Further inquiries can be directed to the corresponding author.

## References

[B1] Agrama H. A. Yan W. Lee F. Robert F. Chen M. H. Jia M. (2009). Genetic assessment of a mini-core subset developed from the USDA rice genebank. Crop Sci. 49, 1336–1346. doi: 10.2135/cropsci2008.06.0551

[B2] Amarnath K. Durga Prasad A. V. S. Chandra Mohan Reddy C. V. (2018). Character association and path analysis in foxtail millet genetic resources (Setaria italica (L.) Beauv. Int. J. Chem. Stud. 6, 3174–3178.

[B3] Anderson T. W. Darling D. A. (1954). A test of goodness-of-fit. J. Am. Stat. Assoc. 49, 765–769. doi: 10.2307/2281537

[B4] Aravind J. Sankar M. S. Wankhede D. P. Kaur V. (2019). Augmented RCBD: Analysis of Augmented Randomised Complete Block Designs (R package version 0.1.1). Available online at: https://aravind-j.github.io/augmentedRCBD/.

[B5] Archak S. Tyagi R. K. Harer P. N. Mahase L. B. Singh N. Dahiya O. P. (2016). Characterization of chickpea germplasm conserved in the Indian National Genebank and development of a core set using qualitative and quantitative trait data. Crop J. 4, 417–424. doi: 10.1016/j.cj.2016.06.013

[B6] Arya R. Bhatt A. Kumar V. Singh P. D. (2017). Correlation analysis of some growth, yield and quality parameters of barnyard millet (Echinochloa frumentacea (Roxb. Link) Germplasm. J. Pharmacognosy Phytochem. 6, 1426–1429.

[B7] Bhatt D. Rasane P. Singh J. Kaur S. Fairos M. Kaur J.. (2023). Nutritional advantages of barnyard millet and opportunities for its processing as value-added foods. J. Food Sci. Technol. 60, 2748–2760. doi: 10.1007/s13197-022-05602-1, PMID: 37711577 PMC10497464

[B8] Brown A. H. D. (1989). Core collections: A practical approach to genetic resources management. Genome 31, 818–824. doi: 10.1139/g89-144

[B9] Burton G. W. (1952). Quantitative inheritance in grasses. Proc. Int. Grassl. Cong 1, 277–283.

[B10] Chandel G. Meena R. K. Dubey M. Kumar M. (2014). Nutritional properties of minor millets: Neglected cereals with potentials to combat malnutrition. Curr. Sci. 107, 1109–1111.

[B11] De Beukelaer H. Davenport G. F. (2018). Corehunter: multi-purpose core subset selection (R package version 3.2.1). Available online at: https://CRAN.Rproject.org/package=corehunter (Accessed July 2024).

[B12] De Beukelaer H. Davenport G. F. Fack V. (2018). Core Hunter 3: flexible core subset selection. BMC Bioinform. 19, 203. doi: 10.1186/s12859-018-2209, PMID: 29855322 PMC6092719

[B13] De Wet J. Prasada Rao K. Mengesha M. Brink D. (1983). Domestication of sawa millet (Echinochloa colona). Econ. Bot. 37, 283–291. doi: 10.1007/BF02858883

[B14] Federer W. T. (1956). Augmented designs Vol. 55 (Hawaiian planter record), 191–208.

[B15] Franco J. Crossa J. Warburton M. L. Taba S. (2006). Sampling strategies for conserving maize diversity when forming core subsets using genetic markers. Crop Sci. 46, 854–864. doi: 10.2135/cropsci2005.07-0201

[B16] Frankel O. H. (1984). “Genetic perspectives of germplasm conservation,” in Genetic Manipulation: Impact on Man and Society. Eds. Arber W. K. Llimensee K. Peacock W. J. Starlinger P. (Cambridge University Press), 161–170.

[B17] Gohel D. S. Chaudhari S. B. (2018). Study of correlation and path analysis of finger millet genotypes (Eleusine coracana L. Gaertn). J. Pharmacognosy Phytochem. 7, 1283–1288.

[B18] Gowda J. Bharathi S. Somu G. Krishnappa M. Rekha D. (2009). Formation of core set in barnyard millet [*Echinochloa frumentacea* (Roxb.) Link] germplasm using data on twenty fourmorpho-agronomic traits. Int. J. Plant Sci. 4, 1–5.

[B19] Gupta A. Mahajan V. Mukesh Kumar M. Gupta H. S. (2009). Biodiversity in the barnyard millet (Echinochloa frumentacea Link, Poaceae) germplasm in India. Genet. Resour. Crop Evol. 56 (6), 883–889. doi: 10.1007/s10722-009-9462-y.

[B20] Hoste I. Verloove F. (2022). Taxonomy of the weed species of the genus Echinochloa (Poaceae, Paniceae) in Southwestern Europe: Exploring the confused current state of affairs. PhytoKeys. 197, 1–31. doi: 10.3897/phytokeys.197.79499, PMID: 36760676 PMC9848994

[B21] Hu J. Zhu J. Xu H. M. (2000). Methods of constructing core collections by stepwise clustering with three sampling strategies based on the genotypic values of crops. Theor. Appl. Genet. 101, 264–268. doi: 10.1007/s001220051478

[B22] Johnson H. W. Robinson H. F. Comstock R. E. (1955). Estimates of genetic and environmental variability in Soybean. Agron. J. 47, 314–318. doi: 10.2134/agronj1955.00021962004700070009x

[B23] Joshi R. P. Jain A. K. Chauhan S. S. Singh G. (2015). Characterization of barnyard millet (Echinochloa frumentacea(Roxb.) Link.) landraces for agro-morphological traits and disease resistance. Electronic J. Plant Breed. 6, 888-898–2015.

[B24] Jyothi T. Kanwar R. R. Shyam K. (2020). Studies of correlation between yield and yield attributing characters of Kodo millet *(Paspalum scrobiculatum* L.). J. Pharmacognosy Phytochem. 9, 1919–1922.

[B25] Jyothsna S. Patro T. S. S. K. Rani S. Y. Ashok S. Neeraja B. Triveni U. (2016b). Studies on genetic variability and inter-relationship between grain yield and its components in foxtail millet (Setaria Italica). Int. J. Agric. Sci. 8(5), 1015–1017.

[B26] Jyothsna S. Patro T. S. S. K. Rani S. Y. Ashok S. Neeraja B. Triveni U. (2016c). Studies on genetic variability and inter-relationship between grain yield and its components in barnyard millet (Echinochloa fruemntacea. Int. J. Agric. Sci. 8, 1012–1014.

[B27] Kaur V. Aravind J. Manju Jacob S. R. Kumari J. Panwar B. S.. (2022). Phenotypic Characterization, Genetic Diversity Assessment in 6,778 Accessions of Barley (Hordeum vulgare L. ssp. Vulgare) Germplasm Conserved in National Genebank of India and Development of a Core Set. Front. Plant Sci. 13. doi: 10.3389/fpls.2022.771920, PMID: 35283876 PMC8913045

[B28] Keuls M. (1952). The use of the “Studentized range” in connection with an analysis of variance. Euphytica 1, 112–122. doi: 10.1007/BF01908269

[B29] Kim K. W. Chung H. K. Cho G. T. Ma K. H. Chandrabalan D. Gwag J. G. (2007). PowerCore: A program applying the advanced M strategy with a heuristic search for establishing core sets. Bioinformatics 23, 2155–2162. doi: 10.1093/bioinformatics/btm313, PMID: 17586551

[B30] Kullback S. Leibler R. A. (1951). On information and sufficiency. Ann. Math. Stat. 22, 79–86. doi: 10.1214/aoms/1177729694

[B31] Kumar S. Ambreen H. Variath M. T. Rao A. R. Agarwal M. Kumar A. (2016). Utilization of Molecular, Phenotypic, and Geographical Diversity to Develop Compact Composite Core Collection in the Oilseed Crop, Safflower (Carthamus tinctorius L.) through Maximization Strategy. Front. Plant Sci. 7. doi: 10.3389/fpls.2016.01554, PMID: 27807441 PMC5069285

[B32] Levene H. (1960). “Robust tests for equality of variances,” in Contributions to Probability and Statistics: Essays in Honour of Harold Hotelling. Ed. Olkin I. (Stanford University Press), 278–292.

[B33] Lush J. L. (1940). Intra-sire correlations or regressions of offspring on dam as a method of estimating heritability of characteristics. J. Anim. Sci. 1940 (1), 293–301.

[B34] Mantel N. (1967). The detection of disease clustering and a generalized regression approach. Cancer Res. 27, 209–220. 6018555

[B35] Marita J. M. Rodriguez J. M. Nienhuis J. (2000). Development of an algorithm identifying maximally diverse core collections. *Genet* . Resour. Crop Evol. 47, 515–526. doi: 10.1023/A:1008784610962

[B36] Maun M. A. Barrett S. C. (1986). The biology of Canadian weeds. 77. Echinochloa crus- galli (L.) Beauv. Can. J. Plant Sci. 66, 739–759. doi: 10.4141/cjps86-093

[B37] Nandini C. Sujata B. Krishnappa M. Aruna Y. R. (2016). Genetic variability, heritability, genetic advance and character association studies in F3generation of cross JK8 x Peddasame (purple late) in little millet (Panicum miliareL. Asian J. Biosci. 11, 244–249. doi: 10.15740/HAS/AJBS/11.2/244-249

[B38] Nanjundan J. Aravind J. Radhamani J. Singh K. H. Kumar A. Thakur A. K. (2021). Development of Indian mustard [Brassica juncea (L.) Czern.] core collection based on agro-morphological traits. Genet. Res. Crop Evol. 69, 145–162. doi: 10.1007/s10722-021-01211-7

[B39] Newman D. (1939). The distribution of range in samples from a normal population, expressed in terms of an independent estimate of standard deviation. Biometrika 31, 20–30. doi: 10.1093/biomet/31.1-2.20

[B40] Odong T. L. Jansen J. Eeuwijk F. A. Hintum T. J. L. (2013). Quality of core collections for effective utilisation of genetic resources review, discussion and interpretation. Theor. App. Genet. 126, 289–305. doi: 10.1007/s00122-012-1971-y, PMID: 22983567 PMC3555244

[B41] Padulosi S. Mal B. S B. R. (2009). Food security and climate change: Role of plant genetic resources of minor millets. Indian J. Plant Genetic Resour. 22, 1–16. Available online at: https://ispgr.in/index.php/ijpgr/article/view/1529.

[B42] Patil H. E. Darjib T. A. Pali V. (2021). Genetic diversity studies for yield and quality traits in barnyard millet. J. Innov. Agric. 8 (1), 21–26. doi: 10.37446/jinagri/rsa/8.1.2021.21-26

[B43] Phogat B. S. Kumar S. Kumari J. Kumar N. Pandey A. C. Singh T. P. (2021). Characterization of wheat germplasm conserved in the Indian National Genebank and establishment of a composite core collection. Crop Sci. 61, 604–620. doi: 10.1002/csc2.20285

[B44] Prabu R. Vanniarajan C. Vetrivanthan M. Gnanamalar R. P. Shanmughasundaram R. Ramalingam J. (2020a). Association studies in barnyard millet (Echinochloa frumentacea(Roxb.) Link) for early maturity and yield contributing traits at high altitude region. Electronic J. Plant Breed. 11 (1), 192–196.

[B45] Prabu R. Vanniarajan C. Vetrivanthan M. Gnanamalar R. P. Shanmughasundaram R. Ramalingam J. (2020b). Diversity and stability studies in barnyard millet (Echinochloa frumentacea(Roxb). Link.) germplasm for grain yield and its contributing traits. Electronic J. Plant Breeding.Vol 11, 528–537.

[B46] Prasanna L. P. Murthy S. J. S. V. Kumar R. P. V. Rao S. V. (2013a). Gene frequency diversity studies in Indian genotypes of Italian millet (Setaria italica(L.)Beauv. Int. J. Curr. Res. 5, 1552–1554. doi: 10.5897/JPBCS2013.0373

[B47] Prasanna P. L. Murthy J. S. V. S. Kumar P. V. R. Rao S. V. (2013b). Nature of gene action for yield and yield components in exotic genotypes of Italian millet. Setariaitalica (L.)Beauv. J. Plant Breed. CropScience 5, 80–84. doi: 10.5897/JPBCS2013.0373

[B48] R Core Team (2024). R: A Language and Environment for Statistical Computing. Vienna, Austria: R Foundation for Statistical Computing. Available online at: https://www.R-project.org/ (Accessed 2024).

[B49] Rao S. S. Agrawal A. P. (2000). Genetic variability, correlation and path coefficient studies in barnyard millet (Echinochloa frumentacea). J. Agric. Sciences. 34(1), 27–31. Available online at: https://www.cabdirect.org/cabdirect/abstract/20053121320.

[B50] Robinson H. F. (1966). Quantitative genetics in relation to breeding of the centennial of Mendalism. Ind. J. Genet. 26, 171–187.

[B51] Saleh A. S. Zhang Q. Chen J. Shen Q. (2013). Millet grains: Nutritional quality, processing, and potential health benefits. Compr. Rev. Food Sci. Food Saf. 12, 281–295. doi: 10.1111/1541-4337.12012

[B52] Shannon C. E. Weaver W. (1949). The Mathematical Theory of Communication (The University of Illinois Press).

[B53] Singh K. P. Mishra H. N. Saha S. (2010). Moisture-dependent properties of barnyard millet grain and kernel. J. Food Eng. 96, 598–606. doi: 10.1016/j.jfoodeng.2009.09.007

[B54] Sivasubramanian S. Madhavamenon P. (1978). Genotypic and phenotypic variability in rice. Madras Agric. J. 60, 1093–1096.

[B55] Smirnov N. V. (1939). Estimate of deviation between empirical distribution functions in two independent samples. Russian). Bull. Moscow Univ 2, 3–16.

[B56] Sood S. Khulbe R. K. Kant L. (2016). Optimal yield related attributes for high grain yield using ontogeny based sequential path analysis in barnyard millet (Echinochloaspp. J. Agr. Sci. Tech. 18, 1933–1944.

[B57] Thachuk C. Crossa J. Franco J. Dreisigacker S. Warburton M. Davenport G. F. (2009). Core Hunter: An algorithm for sampling genetic resources based on multiple genetic measures. BMC Bioinf. 10, 243. doi: 10.1186/1471-2105-10-243, PMID: 19660135 PMC2734557

[B58] Upadhyaya H. D. Dwivedi S. L. Sharma S. Lalitha N. Singh S. Varshney R. (2014). Enhancement of the use and impact of germplasm in crop improvement. Plant Genet. Res. 12, 155–159. doi: 10.1017/S1479262114000458

[B59] Vikram S. Sudhagar R. Masilamani P. Vanniarajan C. (2020). Genetic variability and association analysis in barnyard millet mutants. Electronic. J. Plant Breed. 11.

[B60] Wallace J. G. Upadhyaya H. D. Vetriventhan M. Buckler E. S. Tom Hash C. Ramu P. (2015). The genetic makeup of a global barnyard millet germplasm collection. Plant Genome 8, plantgenome2014–10. doi: 10.3835/plantgenome2014.10.0067, PMID: 33228281

[B61] Wilcoxon F. (1945). Individual comparisons by ranking methods. Biometr. Bull. 1, 80. doi: 10.2307/3001968

[B62] Wilk M. B. Gnanadesikan R. (1968). Probability plotting methods for the analysis for the analysis of data. Biometrika 55, 1–17. doi: 10.2307/2334448 5661047

[B63] Yabuno T. (1971). A note on barnyard millet. Saburo Newslet 3, 43–45.

[B64] Yabuno T. (1987). Japanese barnyard millet in Japan. Econ. Bot. 41, 484–493. doi: 10.1007/BF02908141

