# Supplementary material for: Comprehensive phenotyping of 1,807 Indian barnyard millet (Echinochloa frumentacea Link) accessions from Indian national genebank: unlocking diversity for core set development
Source: Front Plant Sci. 2025 Sep 23;16:1644491. doi: 10.3389/fpls.2025.1644491 (PMC12501797; doi:10.3389/fpls.2025.1644491)
Supplement: Supplementary file 1 [file Table1.docx]

Supplementary Table 1: Descriptors used for characterization of Barnyard millet

| **S.No.** | **Descriptor** | **No.of observation** | **Method of data record** | **Stage of the crop** |
| --- | --- | --- | --- | --- |
| **1** | Growth Habit | Visual observation | 1 – Erect, 2 – Decumbent, 4 - Prostrate | Maximum tillering stage |
| **2** | Early Plant Vigour | Visual observation | 1 - Poor, 2 - Good, 3 - very Good | 2-3 weeks after sowing |
| **3** | Basal Tiller number | 5 random plants | Number of tillers at ground level or from the basal nodes | 40-45 days after sowing |
| **4** | Number of Days to 50% flowering | 5 random plants | No.of days from date of Sowing, Quantitative | Flowering period |
| **5** | Total Number of Leaves | 5 random plants | No. of leaves in a plant Quantitative | Full Foliage stage |
| **6** | Leaf Length (cm) | 5 random plants | Measured of flag leaf in primary tiller, Quantitative | Full Foliage stage |
| **7** | Leaf Width (cm) | 5 random plants | Measured of flag leaf in primary tiller, Quantitative | Full Foliage stage |
| **8** | Leaf Colour | Visual observation | 1 - Pale green, 2 - Light green, 3 - Dark green | Full Foliage stage |
| **9** | Plant pigmentation | Visual observation | 0 – absent, 1 - present | Full foliage stage |
| **10** | Inflorescence colour | Visual observation | 1 – Green, 2 – Light purple, 3 – Dark purple | Reproductive stage |
| **11** | Inflorescence shape | Visual observation | 1 - Cylindrical, 2 - Pyramidical, 3 - Globose-elliptic | Reproductive stage |
| **12** | Inflorescence Compactness | Visual observation | 3 - Open, 5 - Intermediate, 7 - Compact | Reproductive stage |
| **13** | Length of peduncle | 5 random plants | Measured from node to base of inflorescence | Reproductive stage |
| **14** | Plant Height (cm) | 5 random plants | Ground level to the tip of the panicle, Quantitative | Reproductive stage |
| **15** | Length of inflorescence | 5 random plants | Measured from lowest branch to the tip of last branch of the inflorescence | Reproductive stage |
| **16** | Shape of lower racemes | Visual observation | 1 Straight (not slender), 2 Curved (not slender), 3 Slender | Flowering stage |
| **17** | Branching of lower racemes | Visual observation | 0 absent, 1 present | Flowering stage |
| **18** | Spikelet arrangement |  | 1 One side of rachis, 2 arranged around rachis | Flowering stage |
| **19** | Senescence (Stay green trait) | Visual observation | 0-Absent (yellowness at maturity) , 1-Present (Still Green at Maturity) | Maturity stage |
| **20** | Grain Colour | Visual observation | 1 Straw white, 2Grey + Straw white,3 Brownish grey, 4 Grey, 5 Light grey | After harvest of the crop |
| **21** | Grain shape | Visual observation | 1 Concave, 2 Oval | After harvest |
| **22** | Grain yield per plant (g) |  | Mean of 5 plants | After harvest |
| **23** | 1000 grain weight |  | Mean of 5 plants | After harvest |

Supplementary Table 4: Correlation matrix of EC and CS

|  | **TN** | **DFLOW** | **L** | **LL** | **LW** | **LP** | **PH** | **LI** | **1000_GW** | **GYPP** |
| --- | --- | --- | --- | --- | --- | --- | --- | --- | --- | --- |
| **TN** |  | -0.1 | 0.82** | -0.09 | -0.29** | -0.06 | -0.04 | -0.11 | -0.07 | -0.02 |
| **DFLOW** | -0.14** |  | -0.07 | 0.17** | 0.08 | -0.15* | 0.09 | 0.22** | 0.06 | 0.19** |
| **L** | 0.80** | -0.11** |  | 0.08 | -0.14* | -0.07 | 0.15* | 0.01 | -0.06 | 0.08 |
| **LL** | -0.12** | 0.16** | 0.03 |  | 0.57** | -0.08 | 0.42** | 0.50** | 0.05 | 0.09 |
| **LW** | -0.20** | 0.12** | -0.04 | 0.42** |  | -0.04 | 0.44** | 0.31** | 0.06 | 0.13* |
| **LP** | -0.02 | -0.11** | -0.04 | 0.02 | 0.01 |  | -0.12* | -0.02 | 0 | -0.09 |
| **PH** | -0.09** | 0.11** | 0.11** | 0.33** | 0.34** | -0.03 |  | 0.37** | 0.11 | 0.11 |
| **LI** | -0.11** | 0.10** | -0.01 | 0.43** | 0.28** | -0.03 | 0.30** |  | 0.07 | 0.13* |
| **1000_GW** | -0.02 | 0.05* | -0.02 | 0.03 | 0.04 | 0.01 | 0.09** | 0.06* |  | 0.11 |
| **GYPP** | 0.02 | 0.11** | 0.07** | 0.08** | 0.12** | -0.06* | 0.13** | 0.09** | 0.13** |  |
| Below diagonal EC, Above diagonal CS, ** (p = 0.001) Significant | | | | | | | | | | |

Supplementary Table 5: Comparison of various trait variance loadings of entire collection and core set.

| **EC-PC Loadings**` | | | | | |
| --- | --- | --- | --- | --- | --- |
| **Traits** | **PC1** | **PC2** | **PC3** | **PC4** | **PC5** |
| **TN** | 0.329585 | 0.600974 | 0.005908 | -0.02048 | 0.029725 |
| **DFLOW** | -0.24597 | -0.05177 | -0.44707 | -0.28464 | 0.386354 |
| **L** | 0.19521 | 0.67607 | 0.038178 | -0.03942 | 0.031343 |
| **LL** | -0.46461 | 0.193869 | 0.22895 | -0.06909 | -0.00082 |
| **LW** | -0.45582 | 0.106052 | 0.153069 | 0.010098 | 0.105351 |
| **LP** | 0.026062 | -0.06891 | 0.503359 | 0.631129 | 0.477746 |
| **PH** | -0.39652 | 0.247897 | 0.041668 | 0.022389 | -0.10634 |
| **LI** | -0.4168 | 0.166594 | 0.163286 | -0.06358 | -0.22915 |
| **1000_GW** | -0.11082 | 0.05154 | -0.42021 | 0.656688 | -0.52863 |
| **GYPP** | -0.15882 | 0.183402 | -0.51425 | 0.27933 | 0.515998 |
|  |  |  |  |  |  |
| **CS: PC Loadings**` | | | | | |
| **Traits** | **PC1** | **PC2** | **PC3** | **PC4** | **PC5** |
| **TN** | 0.128364 | -0.67538 | 0.002566 | 0.001547 | -0.03883 |
| **DFLOW** | -0.14196 | 0.068235 | -0.50367 | 0.53383 | -0.18706 |
| **L** | -0.02653 | -0.68897 | 0.028328 | 0.009212 | -0.05911 |
| **LL** | -0.51921 | -0.09371 | 0.1582 | 0.076018 | 0.058119 |
| **LW** | -0.47894 | 0.15105 | 0.167799 | 0.018337 | 0.085543 |
| **LP** | 0.100722 | 0.107471 | 0.536883 | -0.0814 | -0.77236 |
| **PH** | -0.45577 | -0.11043 | 0.110099 | -0.06625 | 0.078988 |
| **LI** | -0.45368 | -0.08523 | 0.080786 | -0.00481 | -0.14408 |
| **1000_GW** | -0.10681 | 0.030827 | -0.32497 | -0.83282 | 0.00096 |
| **GYPP** | -0.16914 | -0.03111 | -0.52899 | -0.06473 | -0.57077 |

Supplementary table 2: Promising Barnyard Millet Accessions Across Environments (2023–2025)

| **Trait** | **Promising (Delhi)**  **2023-24** | **Promising**  **(Hyderabad)**  **2023-24** | **Promising**  **(Delhi)**  **2024-25** | **Promising**  **(Jodhpur)**  **2024-25** | **Promising at all Environment (Values in bracket is mean over all location)** |
| --- | --- | --- | --- | --- | --- |
| **Plant height (cm)** | IC0472451  IC0635607  IC0334472  IC0605806  IC0417811  IC0602552  IC0601272  IC0404533  IC0597289  IC0472462  IC0404532  IC0472886  IC0602551  IC0589379  IC0257798  IC0589392  IC0473128  IC0604710  IC0472320  IC0589388  IC0417923  IC0281762.  **(<95 cm)** | IC0629001  IC0417918  IC0637584  IC0472980  IC0472451  IC0589357  IC0472597  IC0610515  IC0589367  IC0472864  IC0589369  IC0472621  IC0268131  IC0472586  IC0473087  IC0589390  IC0340133  IC0472320  IC0417923  IC0472771  IC0589379  IC0255303  **(<50 cm)** | IC0631490  IC0280743  IC0320898  IC0417925  IC0472451  IC0589379  IC0052693  IC0472596  IC0427876  IC0472541  IC0601271  IC0309367  IC0308833  IC0275103  IC0334472  IC0052690  IC0629001  IC0589401  IC0417919  IC0052702  IC0472320  **(<95 cm)** | IC0472320  IC0404300  IC0281486  IC0644179  IC0589379  IC0473138  IC0404530  IC0052693  IC0404345  IC0472720  IC0472451  IC0472708  IC0404475  IC0631490  IC0601265  IC0472977  IC0320898  IC0326016  IC0282780  IC0404368  IC0259309  **(<50 cm)** | IC0472451 (56.86 cm)  IC0589379 (60.44 cm)  IC0472320 (64.33 cm) |
| **Best check value** | Gujrat Banti 1,  (148 cm) | DHBM-93-1  (68.42 cm) | VL-172  (147.17 cm) | DHBM-93-1 (94.19 cm) | Gujrat Banti 1 (125.31 cm)  DHBM-93-3 (118.65 cm)  VL-207 (120.18 cm)  VL-172 (120.70 cm) |
| **Length of Inflorescence (cm)** | IC0279676  IC0472674  IC0472522  IC0273936  IC0417920  IC0281472  IC0279608  IC0472882  IC0472695  IC0472620  IC0404471  IC0472542  IC0610517  IC0404442  IC0472576  IC0281471  IC0406556  IC0472488  IC0355806  IC0473113  IC0279703  **(>30 cm)** | IC0320898  IC0472481  IC0472313  IC0631712  IC0472318  IC0548696  IC0281472  IC0382642  IC0472695  IC0356406  IC0472908  IC0473005  IC0472433  IC0273988  IC0279608  IC0472576  IC0472555  **(>20 cm)** | IC0279608  IC0472708  IC0404404  IC0472916  IC0472576  IC0382642  IC0404362  IC0383387  IC0472600  IC0345054  IC0281456  IC0622126  IC0637913  IC0052690  IC0334067  IC0472543  IC0279676  **(>28 cm)** | IC0279608  IC0628911  IC0472576  IC0472674  IC0391440  IC0404509  IC0618639  IC0472409  IC0383387  IC0599093  IC0268132  IC0473027  IC0472456  IC0473119  IC0404402  **(>27 cm)** | IC0279608 (31.65 cm)  IC0472576 (29.03 cm) |
| **Best check value** | DHBM-93-1  (22.2 cm) | Gujrat Banti 1  (16.06 cm) | VL-172  (22.7 cm) | VL-172  (21.12 cm) | Gujrat Banti 1 (19.33 cm)  DHBM-93-3 (19.27 cm)  VL-207 (17.91 cm)  VL-172 (20.01 cm) |
| **Thousand Grain weight (g)** | IC0306390  IC0473138  IC0510641  IC0472506  IC0598632  IC0404451  IC0548641  IC0404376  IC0444187  IC0404359  IC0120540  **(>4 g)** | IC0306390  IC0643230  IC0597326  IC0404424  IC0404359  IC0472900  IC0404478  IC0345112  IC0308844  IC0472437  IC0472802  IC0052690  **(>4 g)** | IC0629001  IC0404359  IC0306390  IC0052702  IC0363779  IC0309367  IC0345055  IC0404404  IC0326808  IC0472645  IC0052693  **(>4 g)** | IC0404359  IC0306390  IC0472469  IC0097043  IC0472320  IC0473025  IC0393031  IC0472402  IC0472600  IC0472509  IC0404395  **(>3 g)** | IC0306390 (4.71 g)  IC0404359 (4.60 g) |
| **Best check value** | Gujrat Banti 1  (3.04 g) | DHBM-93-1  (3.02 g) | DHBM-93-1  (3.48 g) | Gujrat Banti 1, (2.75 g) | Gujrat Banti 1(2.89 g)  DHBM-93-3 (2.93 g)  VL-207 (2.74 g)  VL-172 (2.70 g) |
| **Days to 50 % flowering** | IC0473119  IC0404530  IC0472916  IC0472491  IC0473061  IC0472553  IC0279792  IC0472387  IC0426592  IC0472899  IC0404294  IC0041794  IC0404300  IC0472354  IC0340124  **(<35 days)** | IC0281441  IC0274183  IC0472767  IC0472418  IC0404300  IC0472852  IC0404302  IC0473002  IC0404530  IC0404294  IC0473064  IC0340124  IC0268135  IC0473070  IC0605806 **(<35 days)** | IC0280742  IC0426592  IC0404530  IC0417919  IC0280743  IC0280746  IC0320898  IC0308833  IC0624715  IC0340124  IC0605806  IC0472320  IC0597324  IC0644179  IC0404300  IC0334472  IC0472746  IC0472859  IC0472891  IC0472509  IC0472451  (<40 days) | IC0340124  IC0472841  IC0426592  IC0469879  IC0404530  IC0472320  IC0404300  IC0472818  IC0472891  IC0120400  IC0383387  IC0589396  IC0052702  IC0404509  IC0472325  IC0472308  IC0472612  IC0279608  IC0605806  IC0308833  IC0472739  **(<40 days)** | IC0404530 (35 days)  IC0426592 (36 days)  IC0404300 (37 days)  IC0340124 (35 days) |
| **Best check value** | VL-172  (52 days) | VL-172  (50 days) | VL-172, (48days) | VL-207  (59 days) | Gujrat Banti 1 (63 days)  DHBM-93-3 (62 days)  VL-207 (56 days)  VL-172 (51 days) |
| **Basal Tiller number** | IC0052702  IC0052699  IC0404366  IC0041791  IC0472857  IC0331100  IC0404320  IC0644179  IC0404520  IC0404467  IC0345146  IC0404317  IC0308843  IC0624707  IC0601265  IC0404504  IC0597324  IC0601275  IC0472641  **(>12 )** | IC0472371  IC0417922  IC0473031  IC0601265  IC0259307  IC0624707  IC0472477  IC0472404  IC0097064  IC0589380  IC0602552  IC0601267  IC0052699  IC0472556  IC0404552  IC0485293  IC0589402  IC0041791  IC0404447  IC0472363  **(>6)** | IC0601265  IC0041791  IC0624707  IC0052693  IC0427868  IC0052702  IC0472541  IC0259313  IC0052690  IC0404300  IC0589375  IC0589379  IC0472609  IC0601273  IC0601174  IC0334022  IC0472556  IC0340124  IC0473092  IC0334472  **(>5)** | IC0624715  IC0624707  IC0601265  IC0426592  IC0309367  IC0472996  IC0417919  IC0308833  IC0472662  IC0041791  IC0472556  IC0637913  IC0334472  IC0120400  IC0589365  IC0472609  IC0605806  IC0597324  IC0472739  IC0472859  **(>6)** | IC0041791 (12)  IC0624707 (13)  IC0601265 (14) |
| **Best check value** | DHBM-93-1,  (5.8) | DHBM-93-1  (3.40) | Gujrat Banti 1 (3.28) | Gujrat Banti 1 (3.48) | Gujrat Banti 1 (3)  DHBM-93-3 (4)  VL-207 (4)  VL-172 (3) |
