# Supplementary material for: Comprehensive phenotyping of 1,807 Indian barnyard millet (Echinochloa frumentacea Link) accessions from Indian national genebank: unlocking diversity for core set development
Source: Front Plant Sci. 2025 Sep 23;16:1644491. doi: 10.3389/fpls.2025.1644491 (PMC12501797; doi:10.3389/fpls.2025.1644491)

Supplementary Figure 1: Boxplot showing comparison of variability of quantitative traits in the Entire collection (EC) and core set (CS) of barnyard millet.


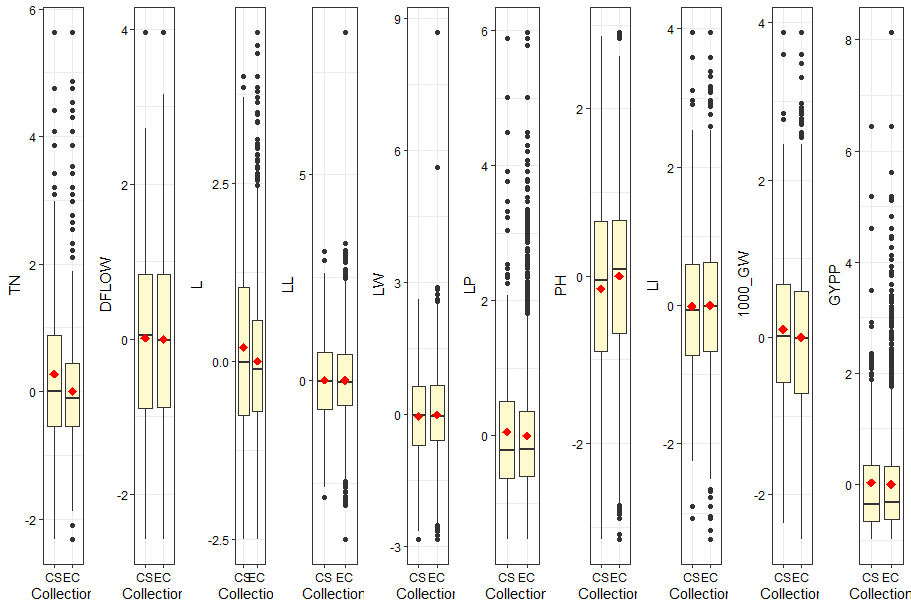


Supplementary Figure 2: QQ plot for Entire Collection vs Core Set


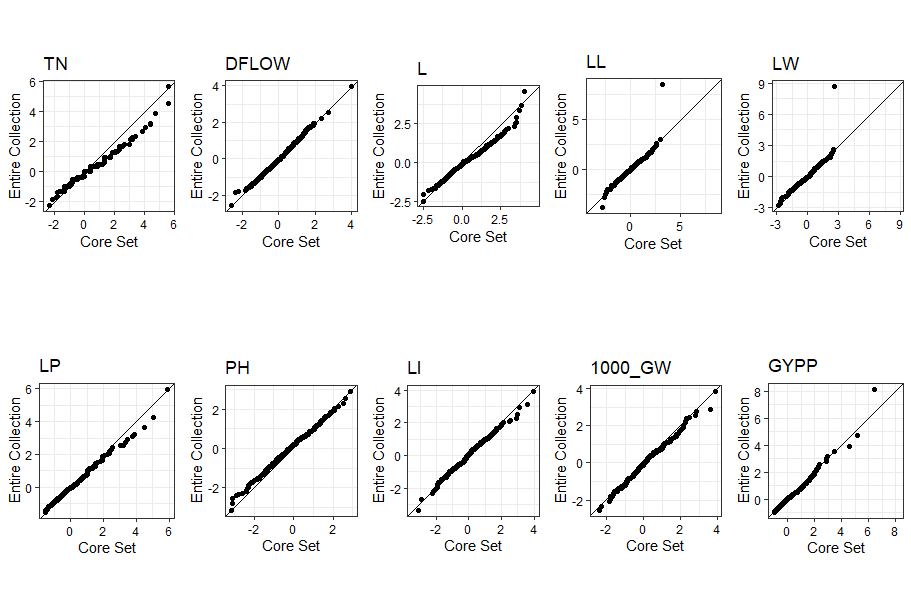


Supplementary Figure 3: Inflorescence variability observed in INGB Barnyard collection.


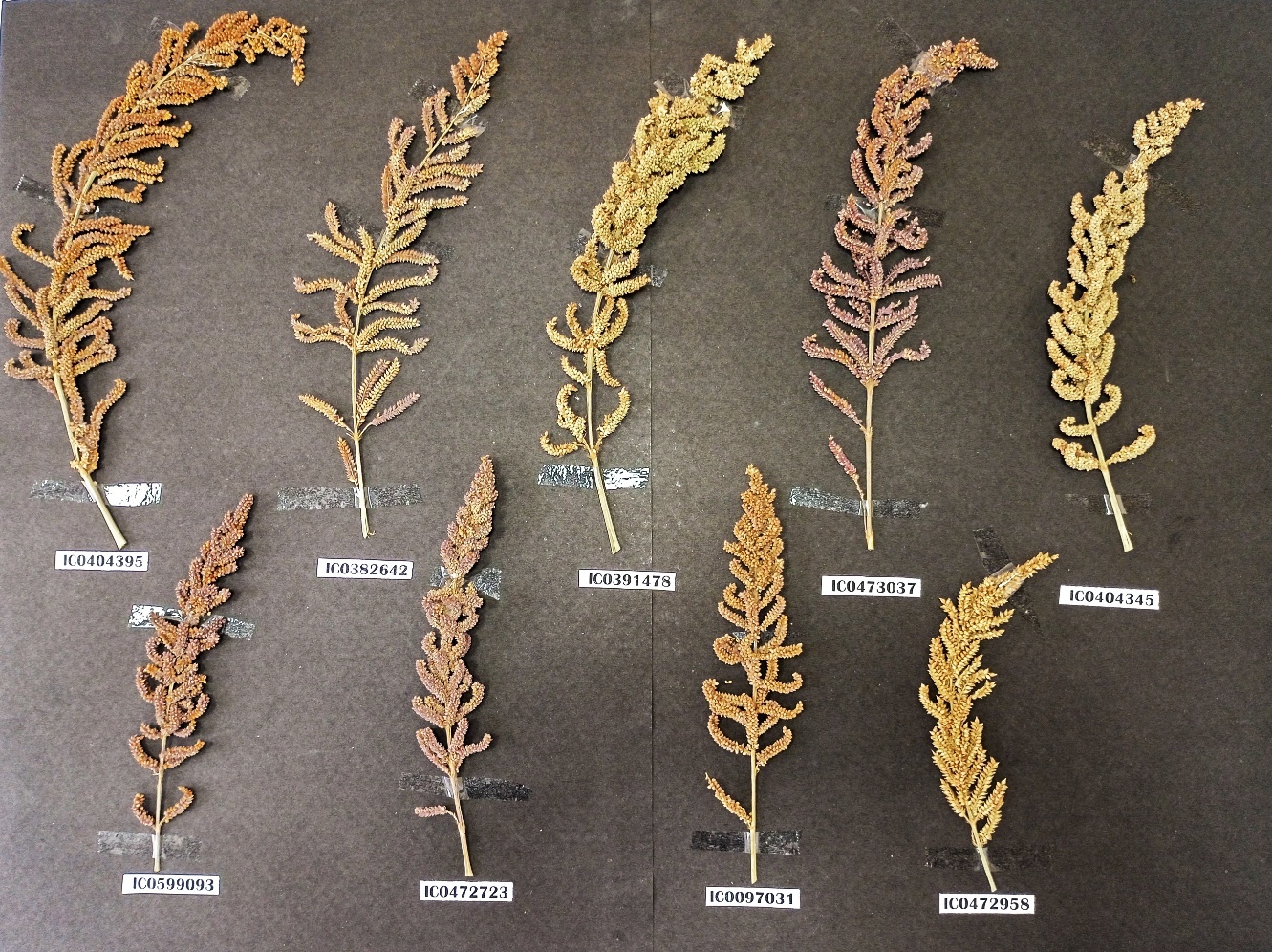


Supplementary Figure 4: Correlation matrix plot based on quantitative traits


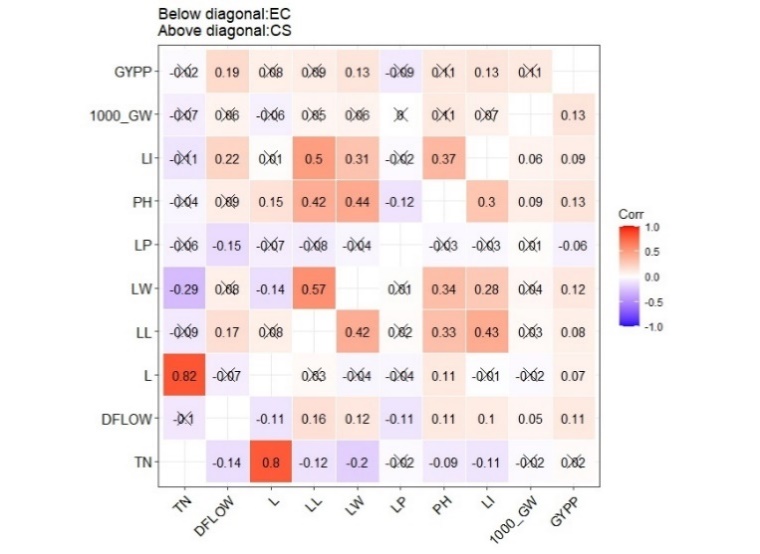


Figure: cross is insignificant at p value 0.001

Supplementary Figure 5: Screeplot and PCA plot for EC and CS


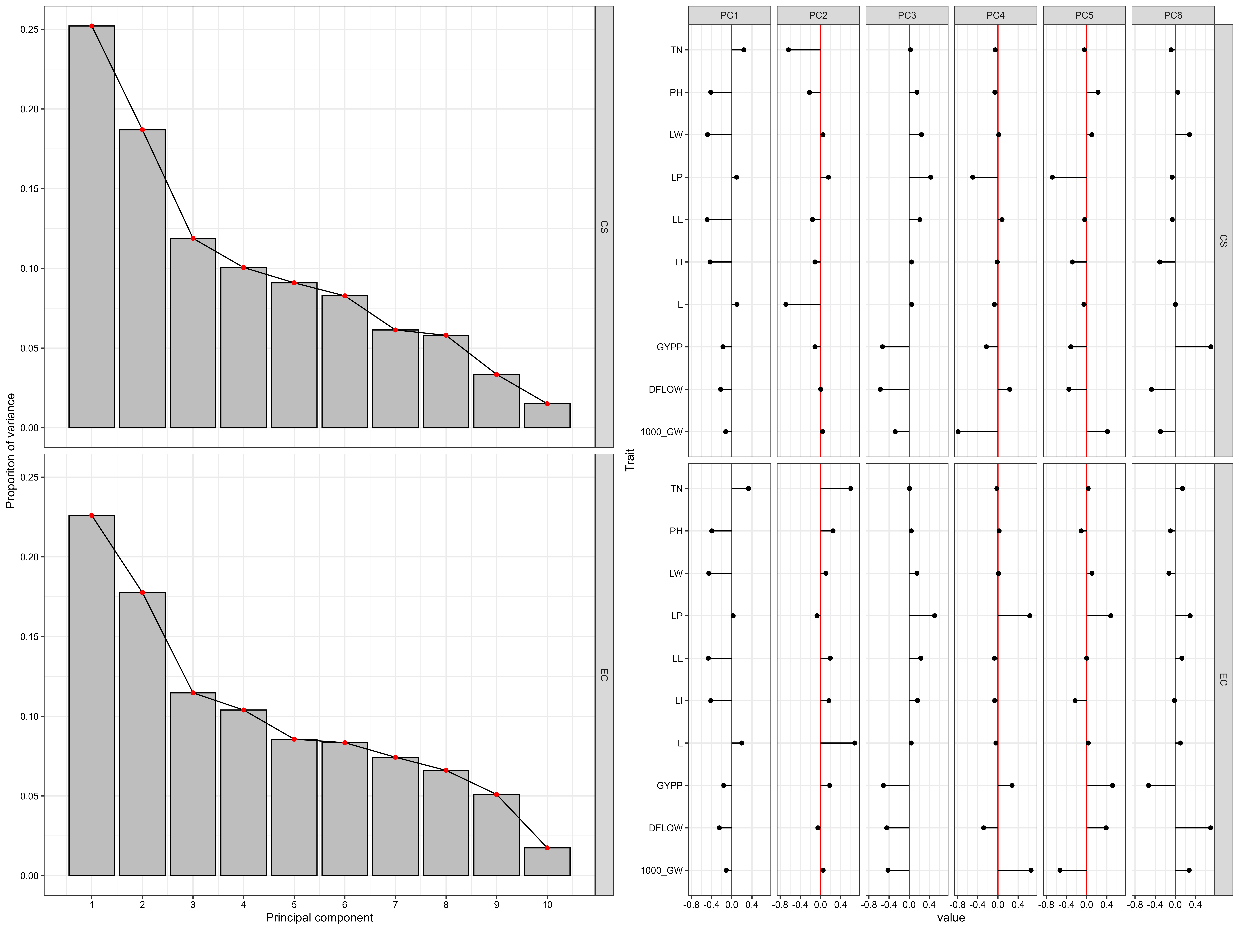

Supplement: Supplementary file 3 [file DataSheet1.docx]
